# Enhancing the Performance of FFF-Printed Parts: A Review of Reinforcement and Modification Strategies for Thermoplastic Polymers

**DOI:** 10.3390/ma18225185

**Published:** 2025-11-14

**Authors:** Jakub Leśniowski, Adam Stawiarski, Marek Barski

**Affiliations:** 1 Laboratory of Techno-Climatic Research and Heavy Duty Machines, CUT Doctoral School, Faculty of Mechanical Engienering, Cracow University of Technology, Al. Jana Pawła II 37, 31-864 Kraków, Poland; jakub.lesniowski@pk.edu.pl; 2 Department of Machine Design and Composite Structure, Faculty of Mechanical Engienering, Cracow University of Technology, Al. Jana Pawła II 37, 31-864 Kraków, Poland; adam.stawiarski@pk.edu.pl

**Keywords:** 3D printing, fused filament fabrication, PLA, ABS, PETG, fiber reinforcement

## Abstract

The technology of 3D printing has become one of the most effective methods of creating various parts, such as those used for fast prototyping. The most important aspect of 3D printing is the selection and application of the appropriate material, also known as filament. The current review concerns mainly the description of the mechanical and physical properties of the different filaments and the possibilities of improving those properties. The review begins with a short description of the development of 3D printing technology. Next, the basic characteristics of thermoplastics used in the fused filament fabrication (FFF) are discussed, namely polylactic acid (PLA), acrylonitrile butadiene styrene (ABS), and polyethylene terephthalate glycol (PETG). According to modern concepts, the printed parts can be reinforced with the use of different kinds of fibers, namely synthetic fibers (carbon, glass, aramid) or natural fibers (wood, flax, hemp, jute). Thus, the impact of such a reinforcement on the performance of FFF composites is also presented. The current review, unlike other works, primarily addresses the problem of the aging of parts made from the thermoplastics above. Environmental conditions, including UV radiation, can drastically reduce the physical and mechanical properties of printed elements. Moreover, the current review contains a detailed discussion about the influence of the different fibers on the final mechanical properties of the printed elements. Generally, the synthetic fibers improve the mechanical performance, with documented increases in tensile modulus reaching, for instance, 700% for carbon-fiber-reinforced ABS or over 15-fold for continuous aramid composites, enabling their use in functional, load-bearing components. In contrast, the natural ones could even decrease the stiffness and strength (e.g., wood–plastic composites), or, as in the case of flax, significantly increase stiffness (by 88–121%) while offering a sustainable, lightweight alternative for non-structural applications.

## 1. Introduction

The advent of additive manufacturing has changed the landscape of production as we know it, with Fused Filament Fabrication (FFF) being one of the most versatile and accessible 3D printing technologies. Also referred to as Fused Deposition Modeling (FDM), FFF works by extruding thermoplastic filaments using a heated nozzle and layering material layer by layer in the fabrication of three-dimensional objects. The technology’s procedural simplicity and low cost have fueled its popularity for applications ranging from rapid prototyping to the direct manufacturing of end-use parts in the automotive, aerospace, and biomedical sectors [1,2,3,4,5,6,7,8,9].

While the intrinsic properties of the feedstock polymer form the baseline for performance, the FFF process introduces significant challenges by creating a unique microstructure. The final mechanical properties are critically dependent on this process-induced structure, which results in significant anisotropy, weak interlayer adhesion, and internal porosity not found in the bulk polymers. Overcoming these process-induced limitations has stimulated voluminous research into material modification and process optimization [3,4,5,6,10,11,12].

Perhaps the most significant of these strategies is the reinforcement of thermoplastics with fibers. The addition of synthetic fibers such as carbon, glass, and aramid has resulted in significantly enhanced tensile properties and stiffness of the FFF composites [3,13,14,15].

In parallel, natural fibers such as wood, flax, jute, and hemp provide environmentally friendly alternatives that have led to a trend to design sustainable composite materials [9,16,17,18,19].

## 2. Search Strategy

The initial search for relevant literature was conducted in the Scopus, Web of Science, and Google Scholar databases. Appropriate keywords were utilized for the identification of sources.

After identifying an initial pool of key articles, snowballing and citation chaining were employed. For each thematic section of the manuscript (e.g., PLA degradation, carbon fiber composites), relevant articles from the initial pool were used as a starting point to identify subsequent, related publications. This process was iteratively repeated to ensure comprehensive coverage of the topic and inclusion of the most recent and influential studies.

Following traditional searching of the references, we also use the AI with a deep search function to find other sources, which can be omitted by traditional browsers. Next, the found items were carefully verified and added to the existing set of references. After that, the process of a deep search has been repeated at least three times per selected topic. It should be stressed here that the AI tool was used only as an auxiliary tool for finding the appropriate references. The date of publication of the selected reference was not limited.

## 3. History and Development of FFF 3D Printing

Fused Filament Fabrication (FFF) is a prominent additive manufacturing technology that has significantly impacted various industries due to its accessibility and versatility [11,20,21,22]. Its development traces back to the late 1980s, laying the groundwork for many of the desktop 3D printers used today [23].

The origins of FDM technology are credited to S. Scott Crump, who co-founded Stratasys Inc. (Eden Prairie, MN, USA and Rehovot, Israel). He patented the Fused Deposition Modeling technique in the late 1980s (U.S. Patent No. 5,121,329, filed in 1989) [21,23,24,25]. A critical component enabling this layer-by-layer construction from digital designs is ‘slicer’ software, which translates the 3D model into machine-readable instructions (G-code) dictating the nozzle’s path, speed, and material extrusion for each layer [21,26,27]. Two years after its founding, Stratasys commercialized the FDM process with their first operational 3D printer, the 3D Modeler, introduced in 1992 [28].

Throughout the 1990s, Stratasys was a key driver in the development of FDM technology. Protected by its foundational patent, the company established it as a major player in the nascent 3D printing market. The company’s FDM printers were notable for their capacity to process a range of industrial-grade thermoplastics; however, achieving consistent reliability across all models presented early developmental challenges [28,29].

**Figure 1:**
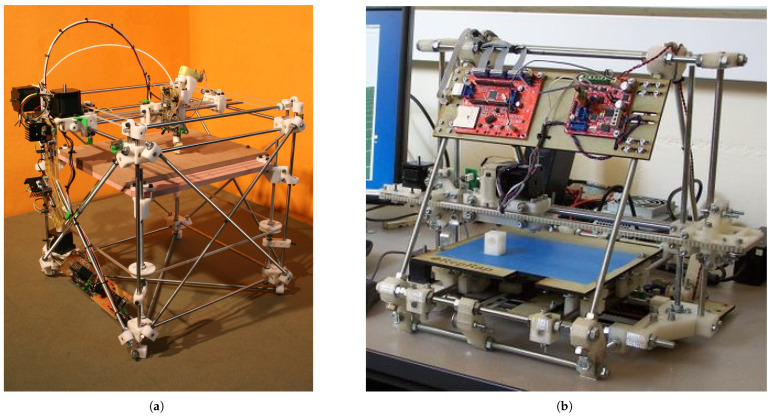
(**a**) The RepRap 1.0 ‘Darwin’ [33]; (**b**) The RepRap 2.0 ‘Mendel’ [35]. These images represent the foundational ‘RepRap’ open-source printers that initiated the democratization and rapid development of FFF technology following the expiration of the FDM patent, enabling community-driven development.

A pivotal moment in the widespread adoption and development of FFF technology occurred with the expiration of the original FDM patent in 2009 [30]. This event democratized the technology, removing a major legal barrier and directly leading to a dramatic decrease in the cost of FFF machines and the proliferation of many new companies entering the market, opening doors for broader commercial use, do-it-yourself (DIY) projects, and the growth of open-source initiatives like the RepRap project, in which hardware designs and software were made publicly available for community-driven development and modification. The RepRap project, aiming to create a self-replicating low-cost 3D printer (meaning a printer capable of fabricating most of its own plastic components), famously initiated by Dr. Adrian Bowyer at the University of Bath, specifically coined the term “Fused Filament Fabrication” (FFF) to provide a legally unconstrained acronym for this type of printing. The success of the RepRap project served as a significant catalyst for the rise of affordable desktop 3D printers and further innovation in the field [24,31,32]. A cornerstone of the open-source movement was the RepRap project’s first successful and publicly documented self-replicating 3D printer, known as RepRap 1.0 “Darwin” shown in Figure 1 [33], whose design was initially released in March 2007 [24,34]. Named after evolutionary biologists, subsequent iterations like the “Mendel” (the second generation design released in 2009) shown in Figure 1 continued to refine the concept, focusing on improved geometries and capabilities, further increasing the accessibility and development of FFF technology [35].

The early 2000s marked a period of continuous improvement in FFF technology, leading to enhanced reliability and increased accessibility for both commercial and personal applications. Advancements in thermoplastic materials and the development of more precise heated nozzles contributed to improved print quality and a wider variety of printable objects. Key hardware advancements that significantly broadened the capabilities and reliability of FFF printers emerged. In the industrial space, enclosed and heated build chambers were crucial for maintaining stable thermal environments when printing temperature-sensitive materials [10,36,37]. In the open-source community, the development of heated build platforms was a critical innovation to improve first-layer adhesion and reduce warping, serving as an effective alternative to patented enclosed chamber designs. The technology transcended its initial prototyping focus to permeate diverse sectors, including medicine and aerospace [20,38].

**Figure 2:**
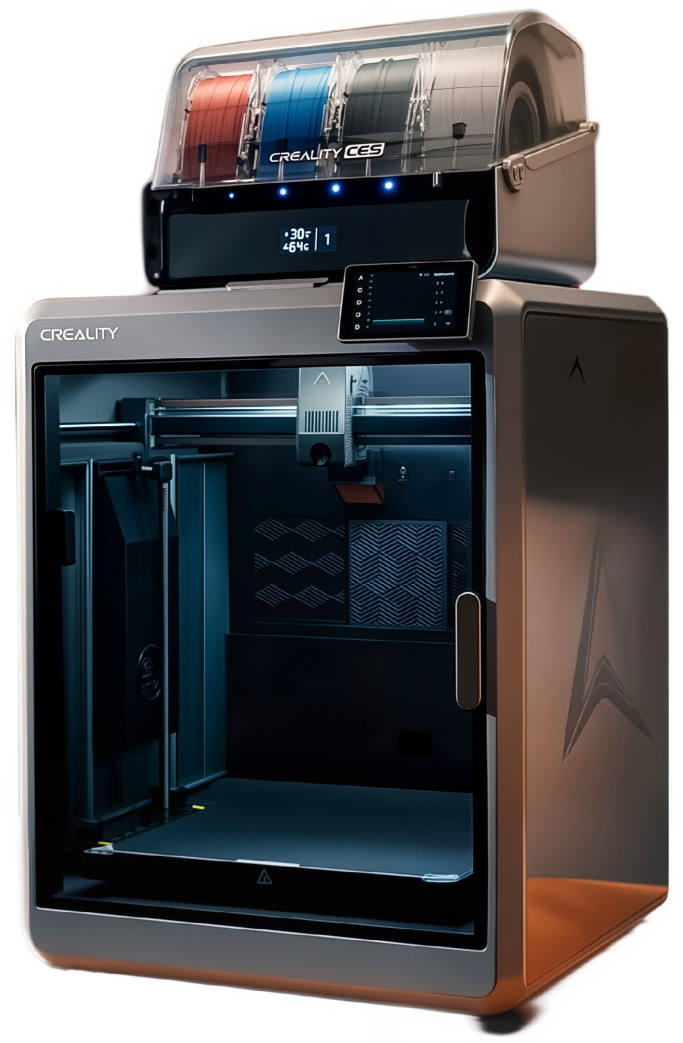
Creality K2 Plus 3D printer. This model exemplifies a key technical conclusion regarding the evolution of FFF hardware: the shift towards high-speed systems (up to 600 mm/s) that significantly reduce production time compared to traditional desktop printers.

More recent developments in FFF technology have focused on increasing print speeds, improving mechanical properties of printed parts, and expanding the range of compatible materials. Innovations such as advanced cooling systems, specialized hotends, and optimized motion control systems have enabled significantly faster layer deposition. The evolution of thermoplastic materials themselves has been critical in advancing high-speed FFF. While traditional desktop FFF printers operated in the range of 50–70 mm/s, as of the year 2025 newer high-speed printers, such as models from Bambu Lab (Shenzhen, China) (e.g., X1 series, P1 series) and Creality (Shenzhen, China) (e.g., K1 series, K2 Plus) shown in Figure 2, can achieve speeds of up to 600 mm/s [39,40,41].

The increasing use of FFF technology in educational institutions, from K-12 schools to universities, also highlights its growing influence and accessibility. Academic institutions have utilized FFF for teaching 3D printing concepts, design skills, and for creating educational aids and assistive technologies. Some universities have even involved students in building 3D printers [20].

FFF technology has evolved from a patented industrial process to a widely accessible and continuously advancing method of additive manufacturing. Key milestones include its invention by Scott Crump, the expiration of the foundational patent leading to market democratization and cost reduction, the rise of open-source initiatives like RepRap initiated by Adrian Bowyer, the development of essential supporting software like slicers, crucial hardware advancements like heated beds and enclosed chambers, and ongoing advancements in hardware, materials, and process parameters aimed at improving speed, reliability, and application diversity [26,28,30,36,39].

A particularly revolutionary future application for additive manufacturing is space exploration. The ability to produce tools and spare parts on-demand in orbit is a key strategy for reducing launch mass and cost, thus enhancing the autonomy of long-term missions. NASA has already successfully tested 3D printers aboard the International Space Station (ISS), manufacturing the first components in microgravity. Future research is expanding beyond polymers to advanced materials like metals and simulated lunar regolith, paving the way for constructing off-world bases from local resources [7,8].

## 4. Basic Characteristics of Thermoplastics Used in FFF

The key mechanical properties of the most widely used thermoplastics in Fused Filament Fabrication Polylactic Acid (PLA), Acrylonitrile Butadiene Styrene (ABS), and Polyethylene Terephthalate Glycol (PETG) have been compiled from the literature and are presented in Table 1.

A characteristic feature of the presented data is the significant range of reported values for each parameter. This variance does not stem from inconsistencies in the feedstock material but is a fundamental consequence of the FFF process itself. This method, through its layer-by-layer deposition of material, creates an anisotropic structure, which directly translates to the anisotropy of the mechanical properties of the final print [3,5,10,42,43].

The upper ranges of these values are typically obtained in tests where the load is applied parallel to the print paths (in the XY plane). In this orientation, the load is borne by continuous strands of the polymer, allowing the material’s full strength to be realized. Conversely, the lower ranges of values are representative of loads applied perpendicularly to the layers (in the Z-direction). In this critical direction, the strength is dictated by the weaker adhesive bonds formed at the interface between layers during thermal fusion. As a result, delamination becomes the dominant failure mechanism [10,43].

This pronounced anisotropy raises a critical question regarding standardized testing methodologies. Currently, the field largely relies on adapting established standards for bulk polymers, such as ASTM D638 [44] for tensile properties and ASTM D790 [45] for flexural properties. However, there is a growing recognition that these classical norms do not fully capture the unique structural characteristics of FFF parts. Consequently, it is considered best practice in the scientific community that a comprehensive report of printing parameters including layer height, build orientation, inf-ill pattern, and density must accompany any reported mechanical data. While dedicated standards for additively manufactured materials are in development, the current practice remains the meticulous documentation of process variables to ensure the reproducibility and comparability of findings.

It is therefore crucial to distinguish that this anisotropy is not an intrinsic property of the polymer itself, but rather a property of the print’s structure, imposed by the manufacturing method. This understanding is paramount for engineers and designers; for any critical application, achieving desired performance requires a general approach that optimizes not only material selection but also strategic part orientation and printing parameters to align the strongest material axes with the principal stress directions in service [3,5].

To illustrate this anisotropic behavior, the data in Table 1 is presented for two common raster orientations relative to the direction of the applied load. A 0° orientation signifies that the print paths are laid down parallel to the testing axis. This configuration generally yields the highest tensile properties, as the load is borne directly along the continuous extruded filaments. In contrast, a ±45° orientation refers to a cross-hatch pattern, where successive layers are printed at +45° and −45° to the loading axis.

**Table 1:** Properties of FFF materials based on print orientation [43,46,47,48,49,50,51,52,53,54,55,56,57].

Material	Tensile Strength (MPa)	Young’s Modulus (MPa)	Elongation at Break (%)	Flexural Strength (MPa)	Extrusion Temperature (°C)	Printing Speed (mm/s)	Layer Height (mm)
*Angle of the print fibers to the test direction: 0°*							
PLA	53–72.3	2451–3769	4.1–5.8	–	200–220	50–70	0.15–0.25
ABS	25–39	1140–1885	3.6–9.5	47	250–255	80–300	0.15–0.20
PETG	33–54	1110–2280	3.2–10.5	–	195–240	50–80	0.20–0.25
*Angle of the print fibers to the test direction: ±45°*							
PLA	48–60	1102–1346	5.2–8.1	97	190–230	40–300	0.20
ABS	31–44	1030–1610	2.8–8.4	–	220–260	65–300	0.15–0.20
PETG	30–51	906–1800	7.4–8.1	35–70	190–270	30–300	0.20

It is crucial to highlight the methodological approach taken in compiling the data for Table 1. A broad, unrestricted survey of existing literature often yields exceptionally wide performance ranges, which can obscure meaningful comparisons. For instance, a less selective compilation might report the tensile strength of FFF-printed ABS as spanning from 11–65 MPa and its Young’s Modulus from 650–2650 MPa. Such a wide statistical scatter is a direct consequence of aggregating results from studies conducted with highly variable and often poorly documented printing parameters. This makes direct comparisons unreliable and hinders the ability to draw substantive conclusions [11,58,59,60,61,62,63,64].

### 4.1. Polylactic Acid (PLA)

Polylactic Acid (PLA), a bio-based aliphatic polyester, stands as one of the most extensively utilized thermoplastics in Fused Filament Fabrication (FFF) 3D printing. Its derivation from renewable resources such as corn starch or sugarcane, coupled with its biodegradability under specific industrial composting conditions, positions it as an environmentally friendlier alternative to many petroleum-derived polymers. The perceived ease of processing, relatively low printing temperatures, and minimal warping during FFF have contributed to its widespread adoption in rapid prototyping, educational settings, and even some end-use applications. This accessibility, particularly on entry-level 3D printers, is a significant factor in its popularity. However, this apparent simplicity at the user level can mask more complex material science challenges related to PLA’s crystallization behavior and thermal properties, which are dynamically engaged during the FFF process. The “ease of printing” often refers to its lower tendency to warp compared to materials like Acrylonitrile Butadiene Styrene (ABS) and its lower printing temperature requirements, but achieving optimal and consistent mechanical properties requires careful control over printing parameters to manage crystallization and ensure robust interlayer adhesion, a non-trivial optimization task.

Key advantages of PLA include its good mechanical strength, particularly stiffness, high dimensional accuracy in printed parts, and a wide range of available colors and grades. Nevertheless, pure PLA also exhibits several limitations that are critical to understand for its effective application and are often the driving force for research into material modification. These include its characteristic brittleness and low toughness, a relatively low glass transition temperature ($T_{g}$), and susceptibility to degradation under certain environmental conditions such as prolonged exposure to moisture, heat, and ultraviolet (UV) radiation. These drawbacks can restrict its use in applications requiring high impact resistance, elevated service temperatures, or long-term outdoor exposure [1,9,65,66].

Furthermore, while PLA’s biodegradability is a significant environmental advantage, its practical degradation often requires specific industrial composting conditions—high temperatures, humidity, and microbial presence—that are not readily available to average consumers or met in typical landfill environments or natural settings where PLA degrades very slowly. This disparity can lead to a “greenwashing” perception if the end-of-life management of PLA products is not properly addressed, thereby impacting its true sustainability profile. The focus on its bio-based origin and biodegradability might overshadow the comprehensive lifecycle aspects, including agricultural inputs for feedstock, energy consumed during polymerization and processing, and the challenges associated with its disposal or recycling [9,66].

#### 4.1.1. Mechanical Characteristics of Pure PLA for FFF Applications

The reported mechanical properties for FFF-printed PLA show a tensile strength between 48–73.2 MPa and a Young’s modulus between 1102–3769 MPa, depending on the print orientation. A critical characteristic of PLA is its limited ductility, which is exacerbated by the FFF process. The material’s elongation at break is consistently low, typically recorded between 4.1% and 8.1%. This brittleness is a primary constraint, as process-induced defects such as microscopic voids and imperfect layer adhesion act as stress concentrators, often leading to premature failure before the bulk material can undergo significant plastic deformation. This limitation has been a significant catalyst for research into material modification strategies aimed at improving toughness, including plasticization, blending, and the development of composites. In terms of flexural performance, which is critical for components subjected to bending stresses, PLA exhibits a flexural strength of 97 MPa. Detailed data on the PLA material can be found in Table 1 [43,46,47,48,53,54].

It should be noted that the mechanical properties and structure of additive-manufactured PLA can be modified via heat treatment after the 3D printing process. This process, which involves heating the parts at specific temperatures (e.g., at 55 °C, 65 °C or 80 °C), can significantly increase tensile strength. Based on the study’s results, the optimal heat treatment process temperature was found to be 65 °C for 5 h. Under this specific heat treatment, the test specimens did not show any deformation, the tensile strength increased by 35%, and the porosity of the PLA structure decreased. Numerous research reports suggest that the reason PLA’s mechanical properties are enhanced as a function of heat treatment is the improvement in the crystallinity of PLA [67].

Given these inherent characteristics, particularly its brittleness and the significant anisotropy introduced by the manufacturing process, pure PLA serves as a crucial baseline material in the context of this analysis. Its well-documented performance, alongside its limitations, provides a fundamental reference point against which the efficacy of various modifications such as blending with impact modifiers e.g., thermoplastic polyurethane (TPU) or ethylene vinyl acetate copolymer (EVA), fiber reinforcement, particulate filling, and plasticization, which will be discussed in subsequent chapters can be systematically evaluated [68,69,70].

#### 4.1.2. FFF Processing Behavior of Pure PLA

PLA is generally processed in FFF at nozzle temperatures ranging from 180 °C to 210 °C. A heated build platform is often recommended for PLA, with typical bed temperatures suggested up to 60 °C (around or slightly below $T_{g}$), to improve first-layer adhesion and reduce the potential for warping. PLA is notably less prone to warping than materials like ABS, partly due to its lower thermal expansion. Print speeds can vary widely depending on the specific printer capabilities and the desired balance between print time and part quality, with common ranges reported between 30 mm/s and 600 mm/s [71,72,73,74,75].

Navigating this optimal processing window represents a non-trivial optimization task, as the final properties of an FFF-printed part are not merely a sum of individual parameter settings but a result of their complex interaction. Factors such as nozzle temperature, print speed, layer height, and cooling rate collectively dictate the thermal history experienced by the material at a micro-scale. This thermal history, in turn, profoundly influences critical aspects like polymer chain diffusion across interlayer boundaries, the degree of molecular orientation, and the development of crystalline structures within the deposited material. Achieving consistent and superior mechanical performance, particularly robust interlayer adhesion and controlled crystallinity, thus hinges on a general understanding and meticulous tuning of these interacting variables to manage the evolution of the material’s mesostructure throughout the FFF process [5,21,76].

The inherently slow crystallization kinetics of PLA can present a challenge in FFF; the rapid cooling experienced by the extruded filament during printing may result in parts that are predominantly amorphous. This tendency towards an amorphous state due to rapid cooling is particularly significant for PLA because its slow crystallization kinetics mean that insufficient time is available for substantial crystalline structures to form during the typical FFF thermal cycle. Unlike some semi-crystalline thermoplastics that can achieve higher crystallinity levels more readily under FFF conditions, PLA often solidifies in a metastable, predominantly amorphous state. This not only affects the initial mechanical properties (e.g., lower stiffness and yield strength compared to a more crystalline counterpart) but also renders the material susceptible to post-processing changes such as physical aging and cold crystallization, potentially leading to dimensional instability and evolving mechanical performance over time. The extent to which this behavior contrasts with other common FFF thermoplastics, and the strategies to manage or leverage PLA’s crystallization behavior, remain important areas for comparative analysis within this meta-analysis. This amorphous state can influence not only the initial mechanical properties but also the post-printing dimensional stability due to phenomena such as physical aging or subsequent crystallization over time or upon exposure to moderate heat [9].

Furthermore, the consistency of the PLA filament diameter (commonly 1.75 mm or 2.85 mm) is a crucial upstream factor that significantly affects extrusion stability and, consequently, the final part quality. Uncontrolled variations in filament diameter can lead to either under-extrusion (resulting in voids and weak interlayer bonding) or over-extrusion (causing dimensional inaccuracies and surface defects), both of which directly compromise the mechanical integrity and precision of the printed component [77,78].

#### 4.1.3. Thermal Aging and Degradation Pathways of PLA

Prolonged exposure of PLA to temperatures such as ambient room temperature can lead to significant thermal aging effects. This aging can manifest as physical aging, a process where the polymer chains in the amorphous regions slowly rearrange themselves towards a more thermodynamically stable, lower-energy (equilibrium) state. Such rearrangements typically result in increased density, stiffness (modulus), and often yield strength, but are commonly accompanied by a decrease in ductility, toughness, and impact strength, leading to increased brittleness. It has been observed that the ductility of 3D-printed PLA specimens stored in the dark at room temperature decreased and subsequently stabilized approximately 5 days after the printing process. This change was attributed to physical aging, a hypothesis validated by differential scanning calorimetry (DSC) tests. This observation implies that the mechanical properties of “freshly printed” PLA parts are not entirely stable and will evolve over a relatively short period even under mild storage conditions, which has significant implications for material characterization protocols (i.e., defining when to test post-printing) and for the predictability of application performance over time [79].

Heat treatment or thermal aging at temperatures above $T_{g}$ (55–65 °C) can also promote further crystallization (cold crystallization) in initially amorphous or semi-crystalline PLA, potentially leading to more significant alterations in mechanical properties such as increased modulus and tensile strength, but often with a further reduction in ductility. The initial degree of crystallinity, influenced by FFF processing, is a key factor. A highly amorphous part, often resulting from rapid cooling during printing, is characterized by a larger volume fraction of material that can undergo physical aging or crystallization upon thermal exposure compared to a part that is already substantially crystalline. Thus, the thermal history imparted during the FFF process, which dictates the initial degree of crystallinity, will strongly influence how the part responds to subsequent thermal aging [9].

#### 4.1.4. UV Radiation Effects (Photodegradation)

Photodegradation is noted as the most common abiotic degradation driving force. In PLA, the effects of photodegradation include an increase in brittleness, crystallinity, and hydrophilicity and a reduction in molecular weight. This process results in a reduction of the polymer’s average molecular weight, which in turn manifests as a loss of mechanical strength, increased brittleness, and often, undesirable aesthetic changes such as discoloration (e.g., yellowing or chalking of the surface). The study showed that although PLA generally experienced fewer chemical and morphological changes compared to ABS after accelerated UV aging, some changes, especially in colorimetric properties, were observed in PLA samples. The extent of photodegradation is dependent on several factors, including the intensity and wavelength of the UV radiation, the duration of exposure, ambient temperature and humidity, and the presence (or absence) of UV stabilizers or certain pigments within the PLA formulation [2,70,80,81,82].

**Figure 3:**
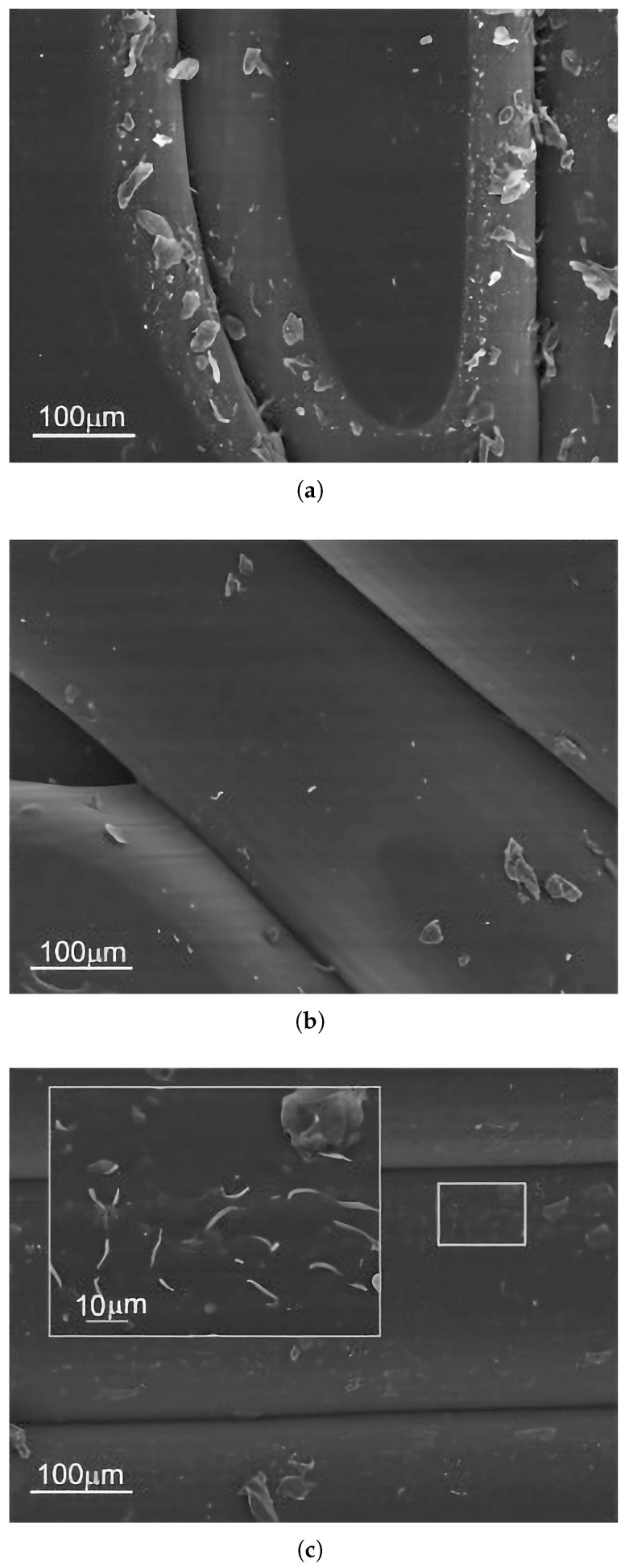
SEM micrographs illustrating the surface morphology of FFF-printed PLA after accelerated aging tests: (**a**) reference sample; (**b**) after 500 h of UV ageing; (**c**) after 500 h of temperature ageing. The images reveal a key technical conclusion: PLA demonstrates high morphological stability under these specific conditions. The surfaces of the aged samples (**b**,**c**) remain smooth and structurally intact, showing no significant evidence of micro-cracking or crazing. This finding is technically important as it suggests that while other properties like color or molecular weight may degrade, the physical surface integrity itself is not the primary failure mode for PLA under these aging regimes [2].

While pure PLA is somewhat susceptible to UV, this degradation mechanism can act synergistically with hydrolytic degradation if moisture is also present, potentially leading to a more rapid overall deterioration of properties in outdoor or humid, sunlit environments. It is also important to consider that the colorants and other additives present even in nominally “natural” or “pure” PLA filaments can significantly influence UV absorption characteristics and the subsequent degradation pathways. This implies that the UV stability of “PLA” is not a uniform characteristic but can vary considerably across different filament brands, colors, and formulations, even if the base PLA resin is similar [70,82,83,84]. Figure 3 provides a visual comparison using scanning electron microscopy, illustrating the surface morphology of a reference PLA sample versus samples subjected to UV and thermal aging.

It is noteworthy that while PLA is susceptible to general UV degradation, its chemical structure shows significant stability against specific high-energy wavelengths. For instance, under controlled UV-C exposure used for sterilization, the tensile and compressive strength of PLA degraded by only 6–8%, a resilience that starkly contrasts with other common thermoplastics [85].

Collectively, these diverse degradation pathways include physical aging, thermal degradation, hydrolysis, and photodegradation have significant implications for the functional lifespan and reliability of PLA components in various service environments. For instance, parts intended for outdoor applications or those exposed to elevated temperatures and humidity will experience accelerated degradation compared to those used in controlled indoor settings. An understanding of these mechanisms is therefore crucial not only for predicting long-term performance but also for informing material selection decisions. When PLA is considered for applications demanding specific durability requirements, its susceptibilities must be weighed against its advantages, or appropriate protective measures or material modifications (such as the incorporation of UV stabilizers or blending with more resistant polymers, as will be explored later) must be considered to ensure fitness for purpose.

### 4.2. Acrylonitrile Butadiene Styrene (ABS)

Acrylonitrile Butadiene Styrene (ABS) is a terpolymer, meaning it is synthesized from three distinct monomers: acrylonitrile, butadiene, and styrene. The specific ratio of these monomers can vary, influencing the final properties of the material, but each contributes uniquely to ABS’s overall performance profile. Acrylonitrile imparts chemical resistance, hardness, and thermal stability; butadiene, a rubbery substance, provides toughness and impact strength, and styrene contributes to the material’s rigidity, processability, and glossy surface finish [86,87,88].

Structurally, ABS is an amorphous polymer, meaning its molecular chains lack long-range order, which contrasts with semi-crystalline polymers like PLA. This amorphous nature means ABS does not have a sharp melting point but rather softens gradually over a temperature range [87,89]

In the context of FFF, ABS offers several key advantages that have cemented its popularity for engineering applications. Its notable mechanical properties, particularly its superior impact resistance, toughness, and good rigidity, make it suitable for producing functional parts and robust prototypes that can withstand mechanical stress. Compared to PLA, pure ABS generally exhibits better thermal stability, allowing components to be used in environments with moderately higher operating temperatures. Furthermore, ABS parts are amenable to various post-processing techniques; they can be easily sanded, drilled, machined, and painted. A significant advantage is its solubility in solvents like acetone, which can be used for chemical vapor smoothing to achieve a very smooth, glossy surface finish or for solvent welding to join multiple printed parts [39,70,88,90].

Despite these benefits, pure ABS presents several inherent limitations and challenges, especially when processed via FFF. Perhaps the most significant challenge is its pronounced susceptibility to warping and cracking during the printing process. This issue arises from its relatively high coefficient of thermal expansion and the consequent shrinkage that occurs as the material cools from extrusion temperature. To mitigate these effects, a heated build platform is almost universally required, and an enclosed print chamber is highly recommended to maintain a stable, elevated ambient temperature around the part during fabrication. Another concern associated with printing ABS is the emission of potentially harmful volatile organic compounds (VOCs), including styrene, which is a known hazardous air pollutant and suspected carcinogen. These emissions necessitate the use of FFF printers in well-ventilated areas or with dedicated fume extraction systems to ensure operator safety and address indoor air quality concerns [4,10,73,91,92]. Beyond general ventilation, the use of enclosed 3D printers, often equipped with integrated air filtration systems (e.g., HEPA and activated carbon filters), is also a common strategy to reduce operator exposure to these emissions.

Environmentally, pure ABS exhibits poor resistance to ultraviolet (UV) radiation. Prolonged exposure to sunlight can lead to significant degradation, manifesting as yellowing, increased brittleness, and a reduction in mechanical properties. While ABS is somewhat hygroscopic, meaning it can absorb moisture from the atmosphere, its tendency to do so it is generally less pronounced than that of materials like nylon or some PETG grades. However, if ABS filaments absorb excessive moisture, it can negatively impact print quality, leading to issues like nozzle sputtering, voids in the printed part, and compromised material properties [53,93,94,95]. Therefore, proper storage in dry conditions or pre-drying of filaments is often advised. Environmentally, beyond UV degradation and hygroscopicity, the end-of-life management of ABS parts also presents challenges. As a petroleum-based thermoplastic, often classified under recycling code 7, has limited municipal recycling.

The performance profile of ABS, offering enhanced mechanical toughness and thermal resistance over materials like PLA, makes it attractive for many engineering-focused applications. However, these advantages are counterbalanced by its more demanding printability requirements and lower environmental stability, particularly concerning UV radiation. This problem underscores a fundamental trade-off in material selection for FFF. The distinct roles of acrylonitrile, butadiene, and styrene in determining ABS’s properties also suggest that modifications to their ratios or chemical structures are primary avenues for developing advanced ABS grades with adjusted characteristics, such as improved UV resistance or enhanced thermal stability [39,53,70,86,88,93]. This understanding of monomer contribution is a cornerstone in polymer science for expected material development.

Typical applications for FFF-printed ABS parts include functional prototypes requiring durability, end-use components subjected to impact or moderate heat (such as custom enclosures for electronics, automotive interior parts, and various consumer products), as well as manufacturing aids like jigs and fixtures where toughness and dimensional stability at slightly elevated temperatures are beneficial [39,87].

#### 4.2.1. Mechanical Characteristics of Pure ABS for FFF Applications

For FFF-printed ABS, the reported tensile strength spans from 25 to 44 MPa. Similarly, the material’s stiffness, quantified by its Young’s modulus, exhibits a wide reported range from 1030 to 1885 MPa. This property is also highly directional and is further influenced by process-dependent factors such as internal void density and the degree of fusion between adjacent beads. A key performance trade-off for FFF-printed ABS is observed in its ductility. While bulk, injection-molded ABS is known for its toughness, the FFF process can induce a more brittle behavior. The reported elongation at break spans a considerable range from 2.8% to 9.5%. The flexural strength of FFF-printed ABS is reported at around 47 MPa. These properties are exceptionally sensitive not only to the build orientation but also to the internal build architecture, including the infill pattern and density. The internal truss-like structures created by different infill strategies significantly alter a component’s response to bending loads, meaning that flexural performance is as much a function of design as it is of the material itself. A summary of key properties for PETG is compiled in Table 1 [49,50,51,53,55,56].

#### 4.2.2. FFF Processing Behavior of Pure ABS

Achieving the desired mechanical properties and overall part quality with Acrylonitrile Butadiene Styrene (ABS) in Fused Filament Fabrication (FFF) is critically dependent on the meticulous control and optimization of various processing parameters. In contrast to materials that are less sensitive to process variables such as PLA, ABS demands a more precise processing window, and deviations can readily lead to common print defects like warping, cracking, and poor interlayer adhesion, ultimately compromising the structural integrity and dimensional accuracy of the printed component [10,56,74,96,97].

The nozzle temperature is a paramount parameter, directly influencing the melt viscosity and the quality of interlayer adhesion. For ABS, the recommended range is 230 °C to 250 °C. Higher temperatures within this window generally improve layer bonding and tensile strength. However, excessively low temperatures can result in insufficient melting and delamination, while exceedingly high temperatures can cause material degradation and loss of dimensional accuracy [42,96,97,98,99,100,101,102].

A heated build platform is necessary when printing with ABS. The recommended bed temperature typically falls within the range of 90 °C to 110 °C. The primary functions of a heated bed is to enhance the adhesion of the first printed layer to the build surface and, crucially, to mitigate warping. By maintaining the base of the printed part at an elevated temperature, closer to its glass transition temperature, the heated bed helps to reduce the thermal gradient between the newly deposited hot material and the already cooled layers, thereby minimizing internal stresses that cause the part to curl or lift at the edges. This thermal management is central to successful ABS printing, as warping is arguably the most significant processing challenge associated with this material due to its high thermal shrinkage [10,39,74,103,104,105].

Print speed is also critical and must be balanced with nozzle temperature. Slower speeds typically promote better layer adhesion, while higher speeds require a corresponding increase in temperature to ensure complete material fusion. Research suggests that combinations of higher nozzle temperatures (e.g., 250 °C) with moderately high print speeds (e.g., 90–110 mm/s) can yield optimal tensile strength for ABS [74,97,98,101,106,107].

Due to its sensitivity to temperature fluctuations and drafts, printing ABS in an enclosed build chamber is highly recommended, and often considered essential for consistent, high-quality results. An enclosure helps to maintain a stable and elevated ambient temperature around the printing part. This controlled thermal environment minimizes rapid or uneven cooling, thereby reducing thermal stresses that lead to warping and cracking, particularly in larger or more complex geometries. Furthermore, an enclosure can improve interlayer adhesion by allowing layers to cool more slowly and uniformly, and it also helps to contain VOC emissions [10,91,108].

#### 4.2.3. Thermal Aging and Degradation Pathways of ABS

ABS is susceptible to degradation when exposed to the high temperatures inherent in the FFF process and during its service life, a phenomenon known as thermal aging. This degradation is primarily a thermo-oxidative process, initiated and accelerated by the combined effects of heat and the presence of oxygen, with mechanical stresses during extrusion also playing a significant role. The resulting irreversible changes to the polymer’s molecular structure can lead to a significant decline in its mechanical properties and aesthetic qualities, manifesting as increased brittleness and discoloration.

The primary site for thermal-oxidative attack in the ABS terpolymer is the polybutadiene (PB) phase, due to the presence of unstable carbon-carbon double bonds in its microstructure. When subjected to heat, these double bonds are susceptible to breaking, which generates active macromolecular radicals. In the presence of oxygen, these radicals initiate an auto-oxidation cycle, a self-propagating chain reaction. Polymer radicals react with molecular oxygen to form peroxyl radicals (representing polymer chains with a peroxide radical group), which in turn abstract a hydrogen atom from another polymer chain to form unstable hydroperoxides (ROOH, representing polymer chains with a hydroperoxide group) and a new free radical, thus continuing the cycle.

The decomposition of these unstable hydroperoxides is the critical step that dictates the degradation pathway, which can proceed via two primary, often competing, mechanisms like chain scission and cross-linking. The balance between these mechanisms is highly dependent on the specific processing conditions. The chain scission process involves the breaking of the main polymer chains, leading to a reduction in the average molecular weight. It is often more dominant under conditions of high mechanical loading, such as the single screw extrusion (SSE) step required to produce filament for FFF. Cross-linking involves the formation of new covalent bonds between adjacent polymer chains, which increases the average molecular weight and can lead to the formation of an insoluble gel fraction. Cross-linking tends to be more prevalent under conditions of high thermal loading with lower mechanical stress.

The most evident macroscopic manifestation of ABS thermal aging is a distinct yellowing of the material. This discoloration is caused by the formation of chromophoric chemical species, particularly carbonyl groups (>C=O), which are byproducts of the oxidation of the PB phase. This change can be quantified using the Yellowness Index (YI). This degradation also impacts mechanical properties; for instance, repeated extrusion cycles can lead to considerable cross-linking, which reduces the melt flow rate (MFR) and can alter the impact strength. These chemical and physical changes can be tracked using various analytical techniques.

Fourier-transform infrared spectroscopy (FTIR) is particularly useful for monitoring the chemical evolution of the polymer by detecting the appearance of new functional groups. During thermo-oxidative degradation, new absorption bands corresponding to hydroxyl (-OH) and carbonyl (>C=O) groups appear, typically around 1720 cm^−1^ for carbonyls. Furthermore, techniques such as size exclusion chromatography (SEC) and rheometry is highly sensitive to changes in the polymer’s average molecular weight and viscosity, allowing for a clear distinction between degradation dominated by chain scission versus that dominated by cross-linking [2,109,110,111].

#### 4.2.4. UV Radiation Effects (Photodegradation) ABS

One of the most significant limitations for the use of pure ABS in outdoor or light-exposed applications is its poor resistance to ultraviolet (UV) radiation. Prolonged exposure to sunlight initiates a process known as photo-oxidative degradation, where high-energy photons provide the activation energy to break chemical bonds and trigger a cascade of deleterious reactions within the polymer.

The underlying chemical mechanism of photodegradation in ABS is closely related to that of thermal oxidation, with the primary difference being the initiation step. Instead of thermal energy, high-energy UV photons break chemical bonds to create free radicals. As with thermal aging, the most susceptible sites are the unsaturated double bonds within the polybutadiene (PB) phase. Once formed, these initial radicals trigger the same auto-oxidation chain reaction involving atmospheric oxygen, leading to the formation of hydroperoxides and subsequent chain scission and cross-linking. A key consequence of this process is the formation of new chemical species, such as carbonyls and hydroxyls, which act as chromophores. These groups absorb light in the visible spectrum, causing the distinct yellowing and discoloration characteristic of weathered ABS.

Direct evidence for this mechanism in FFF-printed parts has been demonstrated in studies where printed ABS samples were subjected to accelerated UV aging. Analysis using Fourier-transform infrared spectroscopy (ATR-FTIR) revealed the appearance of new absorption bands corresponding to hydroxyl ($\nu_{-\mathrm{OH}}$ at $3276\;{\mathrm{cm}}^{-1}$) and carbonyl ($\nu_{-C=O}$ at $1718\;{\mathrm{cm}}^{-1}$) groups. Concurrently, they observed a significant weakening and eventual disappearance of the characteristic absorption bands for butadiene (at $965\;{\mathrm{cm}}^{-1}$ and $910\;{\mathrm{cm}}^{-1}$), providing clear chemical evidence of the preferential photo-oxidation of the PB phase [2,112,113,114,115].

**Figure 4:**
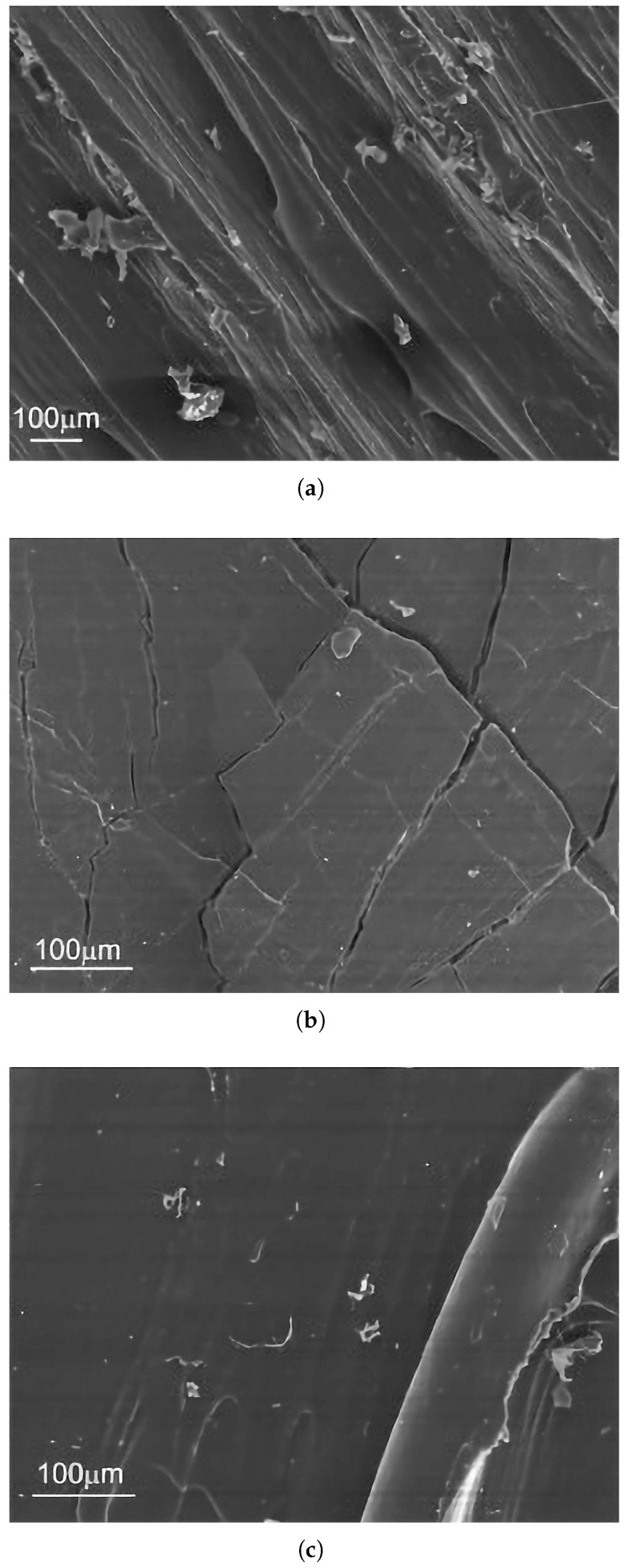
SEM micrographs showing the severe and distinct impact of different aging mechanisms on FFF-printed ABS: (**a**) reference sample; (**b**) after 500 h of UV ageing; (**c**) after 500 h of temperature ageing. The images demonstrate a critical technical conclusion: photodegradation (**b**) causes a dense network of surface micro-cracks, leading to catastrophic brittleness, whereas thermal ageing (**c**) (which causes discoloration) leaves the surface morphology intact. This allows for a clear diagnostic distinction between failure modes [2].

The macroscopic manifestations of photodegradation are profound and multifaceted. The most visually striking effect is the severe degradation of the surface morphology. This phenomenon is clearly illustrated in Figure 4, which provides scanning electron microscopy (SEM) evidence of the material’s surface. The study showed that after 500 h of UV exposure, the initially smooth surface of the ABS sample was covered in a dense network of micro-cracks and crazing. This stands in stark contrast to the thermally aged sample, which showed significant discoloration but remained morphologically intact. This difference in surface morphology provides a powerful diagnostic tool for failure analysis: the presence of extensive micro-cracking is a strong fingerprint of photodegradation, whereas discoloration without cracking is more indicative of thermal aging [114].

In addition to morphological damage, UV exposure causes severe colorimetric changes. The analysis quantitatively documented a progressive and significant yellowing of the ABS samples, confirming the formation of chromophoric degradation products. This surface brittleness and cracking lead to a catastrophic loss of the material’s mechanical toughness. Properties such as impact strength and elongation at break are severely diminished, transforming the once-durable material into a brittle one that is prone to fracture at low strain. While tensile strength may exhibit a smaller reduction, the loss of impact resistance is the defining mechanical failure mode.

Comparative studies have shown that pure ABS is significantly more susceptible to photodegradation than other common FFF polymers like PLA. Under identical accelerated UV aging conditions. ABS underwent severe degradation across all metrics (chemical, morphological, and colorimetric), PLA samples showed very few changes, highlighting the superior intrinsic UV resistance of PLA. To overcome this vulnerability in ABS, industrial strategies typically involve either the incorporation of protective additives, such as UV absorbers or Hindered Amine Light Stabilizers (HALS), or a fundamental change in the polymer chemistry, where the UV-sensitive polybutadiene is replaced with a more stable saturated acrylate rubber [2,114,115].

### 4.3. Polyethylene Terephthalate Glycol (PETG)

Polyethylene Terephthalate Glycol, commonly known as PETG, is a thermoplastic copolyester that has gained significant popularity in FFF for its balanced combination of properties. A modification of Polyethylene Terephthalate (PET), the glycol modification is introduced to inhibit crystallization, which enhances the material’s processability and durability. In the landscape of common FFF materials, PETG is often positioned as a functional intermediate between PLA and ABS, aiming to combine the relative ease of printing associated with PLA with the superior strength and durability characteristic of ABS [116].

Key advantages of PETG include its excellent mechanical strength, superior toughness, and higher impact resistance compared to PLA, making it a more durable choice for functional parts. It is also noted for its good chemical resistance and low thermal shrinkage during the printing process, which results in minimal warping and excellent dimensional stability, often without the need for a fully enclosed build chamber that is critical for ABS. Furthermore, PETG’s high level of optical clarity in its natural form makes it suitable for translucent or transparent applications [53,117].

Despite these benefits, PETG presents its own set of processing challenges. One of the most critical is its hygroscopic nature, meaning it readily absorbs moisture from the atmosphere. Printing with moisture-laden filament can lead to chemical degradation (hydrolysis) at extrusion temperatures, resulting in a visually acceptable but mechanically compromised part with significantly increased brittleness. This makes proper filament storage and drying a non-negotiable prerequisite for reliable performance [118].

Given its favorable balance of printability, mechanical toughness, and chemical resistance, FFF-printed PETG is widely used for functional prototypes, mechanical parts, manufacturing aids, and protective components that require durability and impact resistance beyond what PLA can offer [53].

#### 4.3.1. Mechanical Characteristics of Pure PETG for FFF Applications

The ultimate tensile strength of PETG is reported between 25–54 MPa, while its Young’s modulus spans from 906–2280 MPa. Similarly, flexural properties, with a reported strength of 35 MPa to 70 MPa, demonstrate a strong dependence on process-induced characteristics. As with other FFF materials, a component’s response to bending loads is dictated as much by its internal architecture such as build orientation, infill pattern and density as by the intrinsic properties of the polymer itself. A key advantage of PETG is its superior toughness and impact resistance compared to more brittle FFF materials like PLA. However, its ductility, with a reported elongation at break from 3.2% to 10.5%. The corresponding data for PETG also presented in Table 1 [47,51,52,53,54,57].

Consequently, while PETG is undeniably a tough material, its functional toughness in an FFF context is better characterized by its ability to absorb impact energy rather than its capacity for significant elongation under tensile load [119]. The durability of an FFF-printed PETG part stems not from large-scale plastic deformation, but from its ability to dissipate energy through a cascade of progressive, localized failure mechanisms at the numerous interlayer interfaces, such as micro-delaminations and void deformation [120].

#### 4.3.2. FFF Processing Behavior of Pure PETG

The successful fabrication of high-quality PETG parts is contingent on optimizing a complex window of interdependent FFF processing parameters. Key parameters include nozzle temperature, build platform temperature, and print speed, which must be optimized to balance part strength with print quality [5,75,101,121,122,123].

The recommended nozzle temperature for PETG typically falls within a range of 215 °C to 235 °C. Higher temperatures in this range generally improve interlayer adhesion but can exacerbate PETG’s natural tendency for stringing and oozing due to reduced melt viscosity. A heated build platform is highly recommended to ensure strong first-layer adhesion, with typical bed temperatures set in a range of 70 °C to 90 °C. This helps to counteract the material’s minimal tendency to warp by minimizing thermal gradients. Common print speeds range from 30 to 50 mm/s, although specialized high-speed filaments and printers can achieve much higher rates. Slower speeds are often used to enhance layer fusion, while meticulous tuning of retraction settings is required to mitigate the stringing that can occur, especially at higher temperatures [4,54,75,101,102,121,122,123,124,125,126,127].

Despite its reputation for being relatively easy to print, PETG presents several distinct processing challenges. One of the most critical is its hygroscopic nature; the material readily absorbs moisture from the ambient air. This necessitates storing filament in dry conditions (e.g., a sealed bag with desiccant) and often requires pre-drying the spool in an oven or dedicated filament dryer before printing to achieve optimal results. The consequence of printing with “wet” PETG can be subtle and easily overlooked. While printing with moisture-laden filaments such as ABS or Nylon often produces obvious audible (popping, sizzling) and visual (bubbles, rough surface) defects due to water flashing to steam, PETG’s primary failure mechanism is a more subtle, chemical degradation. At melt temperatures, absorbed water chemically attacks the polyester chains via hydrolysis, breaking them down and reducing the polymer’s molecular weight. This severe degradation can occur without any of the typical signs of a wet filament, allowing an operator to produce a visually perfect and smooth part that is, in fact, chemically compromised, exhibiting significantly increased brittleness and reduced strength. For any engineering application where achieving the intended mechanical performance is essential, filament drying is therefore considered a critical prerequisite for obtaining reliable and consistent results [118,124,128,129,130].

During the high-temperature extrusion process, partial thermal degradation of PETG can occur, leading to the emission of Volatile Organic Compounds (VOCs) and ultrafine particles (UFPs). However, studies have shown that total VOC emission rates from PETG are more than an order of magnitude lower than those from ABS, positioning it as a comparatively lower-emission material. It is important to note, however, that “lower emission” does not equate to “zero emission”. Studies have still identified potentially hazardous VOCs, such as styrene and toluene, being released from PETG during printing, underscoring the continued need for adequate ventilation regardless of the material used [91,131].

Another well-known challenge is PETG’s propensity for stringing (leaving fine plastic hairs during non-extruding travel moves) and oozing from the nozzle. This behavior is linked to its melt viscosity and requires meticulous tuning of retraction settings (distance and speed) and temperature control to mitigate. Finally, while its layer adhesion is generally good, improper settings can still result in internal voids and porosities, and the material can adhere strongly to the nozzle itself, leading to buildup and, in the case of filled variants, accelerated nozzle wear [10,124].

#### 4.3.3. Thermal Aging and Degradation Pathways of PETG

The thermal performance and long-term durability of PETG are primarily dictated by its amorphous nature and its glass transition temperature ($T_{g}$). The $T_{g}$ represents the temperature at which the polymer transitions from a rigid, glassy state to a soft, rubbery state. While reported values can vary depending on the specific material grade and measurement technique, differential scanning calorimetry (DSC) analysis of recycled PETG (R-PETG) has identified the glass transition occurring in a range between 67.5 °C and 77.3 °C. As a material approaches and surpasses this temperature, it begins to soften and lose its structural integrity, particularly when placed under mechanical load [132,133,134,135].

This thermal limitation poses a significant risk for load-bearing PETG components in environments with elevated ambient temperatures, where material softening and accelerated creep can lead to premature failure. This phenomenon implies that a printed component, such as a bracket under a constant load, while stable at room temperature, could exhibit significant creep deformation and potential failure over time if placed in a moderately warmer environment, even at temperatures remaining well below the material’s $T_{g}$. Therefore, for any functional, load-bearing component, the critical design consideration is not simply whether the peak temperature will exceed the $T_{g}$, but rather the interaction between the magnitude of the sustained load, the operational temperature, and the required service life of the part [133].

#### 4.3.4. UV Radiation Effects (Photodegradation) PETG

The resistance of PETG to ultraviolet (UV) radiation is a nuanced topic that highlights the importance of understanding specific environmental stressors. In general comparative literature and for typical outdoor applications, PETG is considered to have good weather resistance. For instance, some studies report that it shows fewer visual signs of degradation, such as yellowing, compared to pure ABS and can even exhibit an increase in tensile strength after weathering. This performance profile makes PETG a suitable material for parts intended for outdoor use [93,136].

This general “UV resistance” does not extend across the entire ultraviolet spectrum. A material’s response to UV radiation is highly dependent on the specific wavelength and energy of the photons. While weathering tests suggest PETG’s performance in natural sunlight is generally robust, it is not immune to photodegradation, with some studies showing notable mechanical property loss under accelerated aging simulating sunlight exposure. For instance, studies using accelerated UV-B aging, which simulates sunlight exposure, have demonstrated a notable 36% reduction in the tensile strength of PETG after 24 h of treatment. However, its degradation becomes exceptionally severe when exposed to high-energy, shortwave UV-C radiation (200–280 nm). This type of radiation is not prevalent in sunlight at the Earth’s surface but is widely used in germicidal lamps for sterilization in medical, laboratory, and industrial settings [85,93,137].

**Figure 5:**
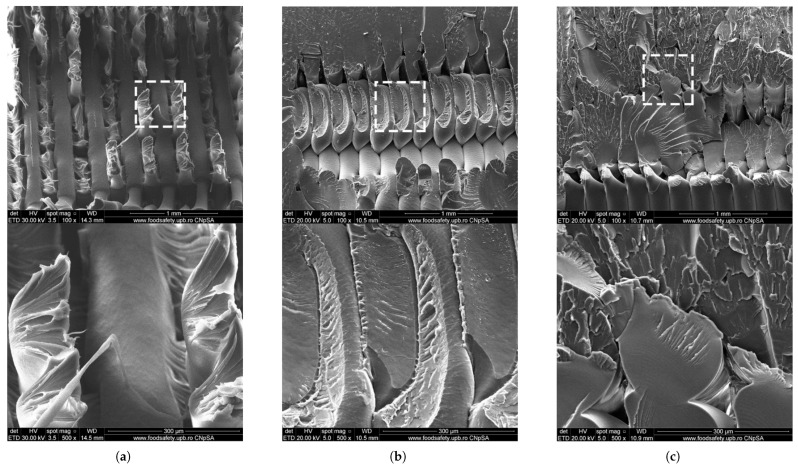
SEM micrographs demonstrating the catastrophic effect of high-energy UV-C radiation on FFF-printed PETG: (**a**) reference sample; (**b**) brittle fracture plane after 24h UV-C exposure; (**c**) severe material flaking at the rupture interface. The main technical conclusion is that “UV resistance” is highly wavelength-specific. While PETG is robust against general weathering (sunlight), it is entirely unsuitable for applications requiring germicidal UV-C sterilization, which causes profound embrittlement and morphological damage, leading to a high risk of premature failure [85].

The effect of UV-C on PETG is exceptionally severe. A study performing accelerated aging with 24 h of continuous UV-C exposure found a profound degradation of mechanical properties in FFF-printed PETG. Specifically, the ultimate tensile strength of the PETG samples was reduced by 38.1%, and their compressive strength was reduced by 33.9%. This dramatic loss of strength and integrity is likely due to extensive chain scission, where the high-energy UV-C photons break the chemical bonds within the polymer backbone [85,138]. The resulting morphological damage, including material flaking and brittle fracture at the rupture interface, is visually documented in Figure 5.

This finding carries profound implications for material selection. The term “UV resistant” is an oversimplification; a material’s performance is a spectrum of vulnerabilities dependent on wavelength. While PETG can be a viable choice for some general outdoor applications, it is entirely unsuitable for components that will be subjected to repeated UV-C sterilization. The use of PETG in such applications would lead to rapid embrittlement and a high risk of unexpected, premature failure. This distinction is essential for engineers designing for advanced applications where environmental conditions, including specific radiation spectra, are precisely defined.

## 5. The Effect of Fiber Reinforcement on the Performance of FFF Composites

The preceding chapters have established the baseline mechanical, thermal, and processing characteristics of the most prevalent pure thermoplastics used in Fused Filament Fabrication (FFF). While materials such as PLA, ABS, and PETG offer significant versatility, their mechanical and thermal limitations often restrict their use to prototyping and low-stress applications. A pivotal strategy to overcome these limitations and elevate FFF-printed parts from conceptual models to functional, end-use components is the incorporation of reinforcing fibers into the polymer matrix. This approach aims to produce polymer matrix composites (PMCs) with adjusted properties capable of withstanding significant mechanical loads in demanding sectors, including aerospace, automotive, and industrial tooling [3,11,139,140].

The field of fiber reinforcement for FFF is broadly categorized into two distinct classes, each driven by different engineering objectives. The first category involves high-performance synthetic fibers, such as carbon, glass, and aramid, which are selected for their exceptional specific strength and stiffness, enabling the production of lightweight, high-strength structural components. The second category comprises natural fibers, including wood, flax, hemp, and jute, which are increasingly investigated for their potential to create sustainable, biodegradable, and cost-effective composite materials [9,139]. This chapter will systematically analyze both categories, beginning with a detailed examination of synthetic fibers, which have set the benchmark for performance in FFF composites.

### 5.1. FFF Composites with Synthetic Fibers (Carbon, Glass, Aramid)

The integration of high-performance synthetic fibers namely carbon, glass, and aramid into thermoplastic matrices represents a transformative step in additive manufacturing, enabling the direct fabrication of functional parts with mechanical properties that significantly surpass those of pure polymers and approach those of conventionally manufactured components. This section provides a systematic analysis of these composites, beginning with the fundamental principles that govern their behavior and performance within the unique constraints of the FFF process. Subsequently, it details the specific characteristics, advantages, and challenges associated with each major fiber type.

#### 5.1.1. Governing Principles of Synthetic Fiber Reinforcement in FFF

The principles of process-induced anisotropy, established for pure thermoplastics in the previous chapter, become even more critical when analyzing fiber-reinforced composites. The addition of fibers transforms the printed part into an engineered mesostructure where performance is dictated not only by layer adhesion but also by the preferential orientation of the fibers along the print path. This added level of controlled anisotropy means that material property data from isotropic bulk samples (e.g., injection molded) is fundamentally unsuitable for predicting the performance of an FFF-printed component [3,5,10,11,22,141].

During extrusion, shear and extensional flows within the nozzle and upon deposition induce a preferential alignment of the fibers along the printing path. This preferential fiber orientation builds upon the anisotropy of the FFF process, further enhancing mechanical properties such as stiffness and strength in the direction of fiber alignment. Consequently, performance in directions transverse to the fibers, and particularly in the inter-layer Z-direction, remains governed by the properties of the polymer matrix and the quality of the fiber–matrix and inter-layer bonds [10,141,142].

This feature of the manufacturing process is not, however, solely a limitation. It can be leveraged as a powerful design tool. Instead of aiming to eliminate anisotropy, which contradicts the physics of the FFF process, the modern approach is to control and utilize it. This concept, known as “anisoprinting,” involves strategically placing fibrous reinforcements along the principal stress directions, allowing for the creation of parts with an exceptional strength-to-weight ratio. This approach elevates the role of the slicer software from a process parameter setting tool to a key element of the composite design itself, where the engineer designs not only the shape but also the internal material architecture [141,143,144,145].

The addition of solid fibers to a molten polymer fundamentally alters its flow characteristics (rheology), primarily by causing a significant increase in melt viscosity. This single change is the root cause of a cascade of interconnected, process-induced defects that can limit the final performance of the composite. The high viscosity hinders the polymer’s ability to flow freely, which simultaneously creates several problems.

First, the stiff, viscous extrudate resists deformation and proper coalescence with previously deposited material, which traps air between adjacent paths and layers and results in performance-degrading voids. At the same time, the sluggish flow prevents the polymer from fully wetting the surface of the reinforcing fibers, leading to weak fiber–matrix interfacial bonding. Finally, the combination of rapid cooling and insufficient flow provides very little time for polymer chains to diffuse and entangle across layer interfaces, causing poor interlayer fusion.

Consequently, mitigating these interconnected defects requires a focus on managing the composite’s rheological behavior. Process optimization strategies typically involve increasing the nozzle temperature to reduce viscosity or decreasing the print speed to allow more time for flow, diffusion, and fusion. Furthermore, while the extrusion process itself has been found to contribute very little to fiber breakage, the quality of the interfacial bond between the fibers and the polymer matrix emerges as a prominent failure mechanism in the final composite [10,22,141,142,146,147,148].

#### 5.1.2. Carbon Fiber (CF)-Reinforced Composites

**Figure 6:**
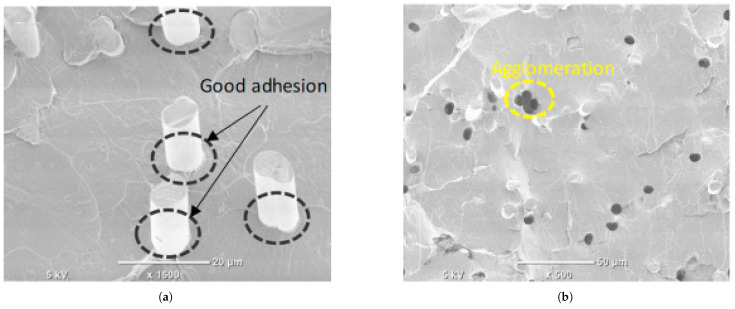
SEM cross-sections of a PLA/rCF composite filament illustrating the two critical microstructural factors governing mechanical performance. (**a**) Desired state: evidence of good fiber–matrix adhesion, which is essential for effective stress transfer from the polymer to the stiff fibers. (**b**) Common defect state: fiber agglomeration (clumping). The technical conclusion is that composite strength is dictated by this micro-scale quality; agglomerations (**b**) act as stress concentrators and weak points, undermining the reinforcing potential of well-bonded fibers (**a**) [152].

Carbon fiber (CF) is regarded as a leading reinforcement material for advanced FFF applications due to its exceptional specific stiffness (stiffness-to-weight ratio), high specific strength, and excellent thermal and chemical stability. The addition of chopped carbon fibers dispersed in the filament significantly enhances the stiffness (Young’s modulus) of common thermoplastics and improves their dimensional stability by reducing thermal warping during the printing process. Studies have reported dramatic improvements in tensile modulus for parts printed along the fiber orientation, with increases ranging from over 160% for PLA-CF to 313% for PETG-CF and as high as 700% for ABS-CF compared to their unfilled counterparts. While strength is markedly increased along the fiber alignment axis, this effect is highly anisotropic. For parts printed with a 0° raster angle, tensile strength can increase by 14–47% for PLA-CF, 22.5–33% for ABS-CF, and 48% for PETG-CF Critically, studies have shown that with improper fiber orientation, such as transverse to the load axis (e.g., 90°), the tensile strength of some carbon fiber composites can fall below that of the unreinforced polymer, with reported decreases of up to 15.7% for ABS-CF, a key design consideration for functional components [60,139,140,142,143,149,150,151]. At the microstructural level, the effectiveness of this reinforcement depends on factors such as fiber–matrix adhesion and the avoidance of fiber agglomeration, as illustrated in the SEM images in Figure 6.

Equally important is the improvement in thermal stability. The low coefficient of thermal expansion (CTE) of carbon fiber is particularly advantageous in FFF, as it limits the warping tendency of high-shrinkage polymers like ABS, enabling the printing of larger and more dimensionally accurate parts. Furthermore, CF reinforcement significantly raises the heat deflection temperature (HDT), expanding the material’s operating temperature range [139,140,142,151,153].

The biggest challenge specific to CF composites is extreme nozzle abrasion. Carbon fibers are exceptionally hard and cause rapid wear of standard brass nozzles, a well-documented phenomenon in FFF. This wear gradually enlarges the nozzle orifice, leading to a loss of dimensional accuracy, inconsistent extrusion, and a deterioration of mechanical properties over the course of a single or multiple prints. Therefore, the use of wear-resistant nozzles (e.g., made of hardened steel, ruby, or silicon carbide) is an absolute prerequisite for reliable and repeatable printing of CF-filled materials [154].

The economic analysis extends beyond the simple price of the filament, which, while significantly higher than standard thermoplastics, is only one component of the “total cost of ownership”. This comprehensive cost includes recurring capital expenditures for expensive, wear-resistant nozzles, which must be treated as consumable items with a finite lifespan due to the abrasive nature of carbon fibers. Furthermore, the risk of print failure due to process instabilities, such as under-extrusion caused by nozzle wear, introduces a significant financial risk from wasted material, machine time, and labor. Consequently, the economic barrier to entry for reliable CF composite printing is higher than the filament cost alone would suggest, requiring a thorough cost-benefit analysis that accounts for these process-specific operational expenses [139,155].

Beyond structural reinforcement, the intrinsic electrical conductivity of carbon fiber enables the fabrication of multifunctional composites with integrated capabilities, such as for electromagnetic interference (EMI) shielding, piezoresistive strain sensing for structural health monitoring, and as electrodes for energy storage devices [140,156].

#### 5.1.3. Glass Fiber (GF)-Reinforced Composites

Glass fiber (GF) reinforcement represents a highly effective method for improving the mechanical properties of FFF-printed thermoplastics and is widely regarded as a more economical alternative to carbon fiber reinforcement. It is positioned as a pragmatic alternative to carbon fiber, offering an attractive compromise that typically trades the superior stiffness and lower weight of CF for the enhanced toughness, impact resistance, and significantly lower cost of GF composites, making them suitable for a wide range of industrial applications [139,157].

**Figure 7:**
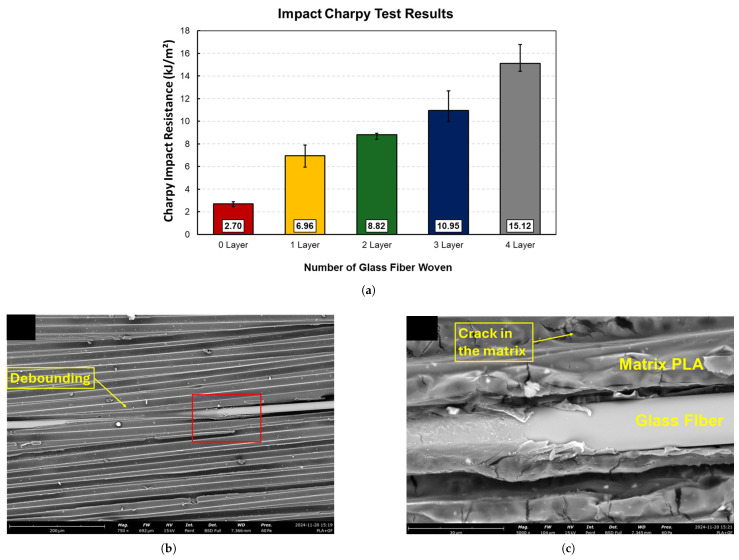
The results of the impact test on PLA+ composites reinforced with woven glass fiber and morphology analysis using SEM: (**a**) graph impact resistance, (**b**) morphology at magnification 750× magnification (red box indicates the area magnified in subfigure c), (**c**) morphology at 5000× magnification. The key technical conclusion from (**a**) is that Charpy impact resistance increases (over 460% improvement) with each added layer of glass fiber. The SEM images (**b**,**c**) reveal the energy absorption mechanisms, such as fiber debonding and matrix cracking, which contribute to this increased toughness [161].

While it does not achieve the same high stiffness as CF, it provides a significant improvement in strength, stiffness, and impact resistance compared to pure polymers. The addition of short glass fibers (SGF) has been shown to increase the tensile strength of ABS composites by 31–57% and the tensile modulus by up to 68%. For SGF-reinforced PLA, tensile strengths of approximately 49.6 MPa have been reported, representing a notable increase over many neat PLA formulations. Studies show that the addition of 15–30 wt% SGF to an ABS matrix can increase flexural properties by 44–59%. A key advantage over CF is often its superior impact resistance and a more ductile failure mode. The addition of SGF to an ABS matrix has been shown to increase Izod impact strength by up to 54%. This effect is even more pronounced with continuous fibers; studies using woven glass fiber in a PLA+ matrix demonstrated exceptional failure resistance, increasing the Charpy impact energy absorption from 2.70 kJ/m^2^ to 15.12 kJ/m^2^, a 460% improvement [157,158,159,160,161]. This enhancement in impact performance, along with the corresponding failure mechanisms such as fiber debonding and matrix cracking, is demonstrated in Figure 7.

This combination of lower cost and significant property enhancement positions glass fiber as a key material for the broader industrial adoption of functional FFF composites. While the high material and process costs of carbon fiber often limit its use to high-performance sectors like aerospace, glass fiber offers a sufficient property uplift for a wider spectrum of applications. The orthotropic nature of GF-printed parts is so pronounced that Classical Lamination Theory (CLT), a tool from traditional composite engineering, has been successfully applied to model their behavior. Additionally, some studies focus on the long-term performance of GF composites in environmental conditions, analyzing the impact of moisture and temperature on their mechanical properties [10,14,139,157,162,163].

#### 5.1.4. Aramid Fiber-Reinforced Composites

The main advantage of aramid is its ability to impart significant toughness and resistance to impact, which results in high energy absorption and a ductile, non-catastrophic failure mode. In contrast to the brittle fracture characteristic of many carbon fiber composites, parts reinforced with aramid exhibit a more ductile failure mode; they tend to bend and deform rather than fracturing abruptly. This failure characteristic suggests its suitability for applications requiring high durability and damage tolerance, such as components for robotic end-of-arm tooling or protective gear. Studies on continuous aramid fiber composites demonstrate a dramatic improvement in mechanical properties over pure polymers; for instance, reinforcing PETG with 45 vol% of aramid fiber can increase the tensile modulus by over 15-fold (from 2200 MPa to 33,000 MPa), tensile strength by over 10-fold (from 42.3 MPa to approx. 450 MPa) and flexural modulus by nearly 17-fold (from 1600 MPa to 27,000 MPa). Hybridization of composites with aramid fibers has been shown to improve energy absorption. Research indicates that hybrid PLA composites containing both carbon and aramid fibers absorb between 5.52% and 11.64% more energy under impact compared to non-hybrid composites reinforced only with carbon fiber [164,165,166,167].

Perhaps the most significant processing challenge for aramid-reinforced nylon composites is their pronounced hygroscopicity. This issue is compounded by the fact that both the aramid fibers and the commonly used polyamide (nylon) matrix readily absorb moisture from the ambient environment. This creates a synergistic problem: during high-temperature extrusion, trapped moisture vaporizes, leading to two distinct but coupled destructive effects. First, the rapid expansion of steam creates voids and porosity within the printed part, compromising its structural integrity. Second, the hot water vapor can chemically attack the polyamide chains via hydrolysis, causing chain scission that reduces the matrix’s molecular weight and intrinsic mechanical strength. Consequently, a failure to rigorously manage moisture leads not only to poor print quality but to a fundamental degradation of both the matrix and the fiber–matrix interface. This elevates the role of auxiliary equipment and procedures such as dedicated filament dryers and moisture-controlled storage from best practice to an absolute prerequisite for achieving reliable mechanical performance [15,168].

**Figure 8:**
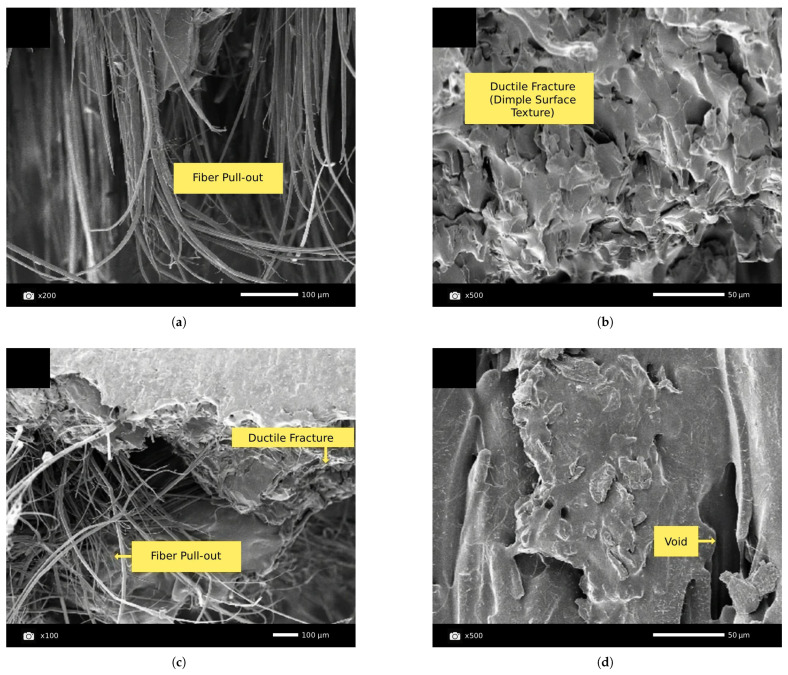
SEM analysis revealing the complex, multi-stage failure mechanisms that define the high toughness of aramid-reinforced composites. The main technical conclusion is that high energy absorption is achieved through a combination of (**b**) ductile fracture (dimple surface texture) of the matrix and (**a**,**c**) extensive fiber pull-out. While this pull-out contributes to a non-catastrophic failure, it also (**a**) highlights the key technical challenge of poor interfacial adhesion between the aramid fiber and the matrix. (**d**) Process-induced voids are also visible, which can act as failure initiation sites [169].

An additional challenge is achieving a strong interfacial bond between the chemically dissimilar aramid fiber and the thermoplastic matrix, which can lead to failure modes such as delamination and fiber pull-out, thereby limiting the full utilization of the reinforcement’s potential [147,167]. Figure 8 illustrates these complex failure mechanisms at the microstructural level, showing characteristic fiber pull-out resulting from poor adhesion, alongside evidence of ductile fracture in the matrix, which contributes to the material’s overall toughness.

Despite this challenge, studies show that even low fiber volume fractions can yield significant improvements; for example, Onyx™-Aramid composites with less than 19 percent by volume of fibers have demonstrated a four-fold increase in tensile modulus over the neat matrix. Recent work has focused on adapting low-cost, desktop printers to produce high-performance aramid/PETG composites, achieving very high fiber volume fractions (up to 45 vol%) through in-nozzle impregnation techniques [167,170].

#### 5.1.5. Comparative Analysis and Overarching Challenges

This final section reviews publications that directly compare fiber types or address universal challenges in producing FFF composites, making the trade-offs explicit. The choice of a synthetic fiber for reinforcing thermoplastics in FFF involves balancing specific performance attributes, costs, and processing requirements. Each of the three primary synthetic fibers carbon, glass, and aramid occupies a distinct position in this design space [139,141,171].

In terms of tensile performance, continuous CF-reinforced composites fabricated by FFF consistently demonstrate the highest specific stiffness and strength, significantly outperforming their GF and aramid counterparts across various thermoplastic matrices. This hierarchy of tensile performance, however, is inverted when considering toughness and impact resistance. Aramid fibers, such as aramid, are renowned for their exceptional energy absorption and a ductile, non-catastrophic failure mode, which involves significant fiber pull-out and delamination rather than abrupt fracture. This stands in stark contrast to the brittleness of CF composites. Glass fiber also provides excellent impact resistance, often serving as a cost-effective alternative to aramid. Notably, some studies indicate that under certain loading conditions, GF composites can exhibit impact energy absorption comparable or even superior to their aramid counterparts, positioning both as primary choices for applications requiring high damage tolerance [166,172,173,174,175,176]. In terms of thermal stability, carbon fiber composites are known for their very low coefficient of thermal expansion (CTE), which contributes to high dimensional stability during printing, and they also typically exhibit a high heat deflection temperature (HDT). Cost is a defining factor in fiber selection: glass fiber is by far the most economical synthetic reinforcement, while carbon fiber is the most expensive, creating a wide cost-performance spectrum. Aramid fibers represent a high-cost specialty option, priced significantly above glass fiber. To achieve a more balanced performance profile, some studies explore hybrid systems, such as combining carbon and aramid fibers to leverage the high stiffness of carbon alongside the exceptional toughness of aramid [140,166,172,177].

A key, overarching challenge is the persistent performance gap between FFF-printed composites and those produced by traditional methods. This is due to the lower achievable fiber volume fractions, process-induced voids, and weaker interlayer bonds in FFF. The literature points to a multi-faceted approach to closing this gap, involving hardware innovations (better extruder/nozzle designs for impregnation, in-process compaction, post-processing in an autoclave) and material innovations (new polymer matrices, specialized fiber surface treatments) [11,22,141,171,178,179,180,181].

To synthetically summarize and directly compare the discussed materials, Table 2 compiles the key properties, advantages, and primary challenges associated with the use of synthetic fiber-reinforced composites.

### 5.2. FFF Composites with Natural Fibers (Wood, Flax, Hemp, Jute)

The integration of natural fibers into thermoplastic matrices for FFF applications represents a significant paradigm shift in materials science, driven by a growing demand for sustainable, renewable, and biodegradable alternatives to conventional petroleum-derived polymers and synthetic reinforcements. The utilization of natural fiber composites (NFCs) aligns with the principles of a circular economy by leveraging low-cost, lightweight, and readily available resources, many of which are byproducts or waste streams from agriculture and forestry. In the context of FFF, the most commonly investigated natural fibers include wood flour and bast fibers such as flax, hemp, and jute. These materials offer the potential to enhance certain mechanical properties, particularly stiffness, while also reducing the overall material cost of the filament feedstock. However, the widespread adoption of NFCs in FFF is hindered by a set of fundamental and interconnected material science challenges that dominate the research landscape [65,182,183,184,185]. These can be categorized into three primary areas. First is the hydrophilicity of natural fibers, their strong chemical affinity for absorbing moisture from the environment stands in stark contrast to the hydrophobic nature of common thermoplastic matrices like PLA. This mismatch leads to significant issues with dimensional stability and long-term performance degradation in humid conditions [186,187]. Second is the poor thermal stability of lignocellulosic materials. The constituent components of natural fibers, particularly hemicellulose and lignin, begin to degrade at temperatures above approximately 200 °C to 220 °C, which severely constrains the viable processing window for FFF and limits the selection of compatible high-temperature polymer matrices [184]. Third, and perhaps most critically, is the weak interfacial adhesion between the hydrophilic fibers and the hydrophobic polymer matrix. This chemical incompatibility results in a poor bond at the fiber–matrix interface, which compromises the ability to effectively transfer stress from the matrix to the stronger fibers and often leads to a reduction in the composite’s mechanical strength compared to the pure polymer. Consequently, much of the research and development in the field of FFF-printed NFCs is dedicated to addressing these three core challenges [65,188,189].

#### 5.2.1. Wood–Plastic Composites (WPCs)

**Figure 9:**
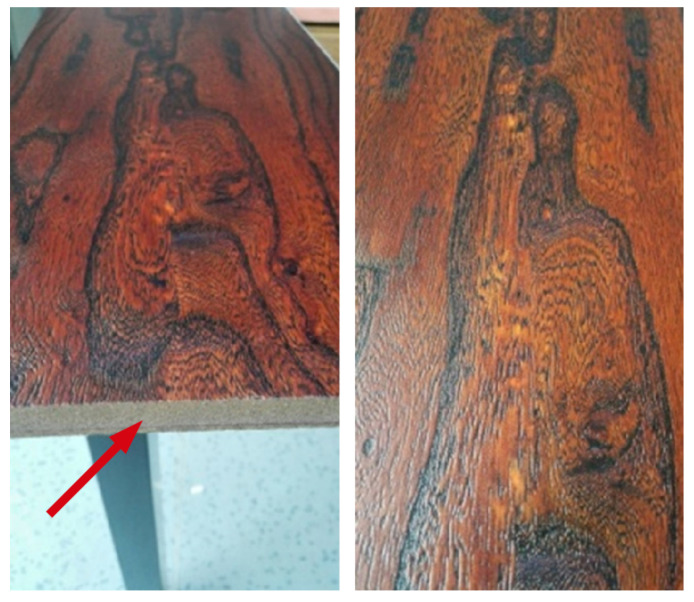
An example of a primary application for WPCs: direct-to-substrate 3D printing to create a realistic elm wood texture. The red arrow indicates the substrate material onto which the texture is printed. The technical conclusion this illustrates is that the main engineering driver for WPC use is often aesthetic and cost-reduction, rather than structural reinforcement. Research consistently shows that wood flour typically acts as a filler, which can significantly reduce mechanical strength compared to the neat polymer. This positions WPCs as an ideal material for non-structural, decorative applications like the one shown [192].

Wood–Plastic Composites (WPCs), which typically utilize fine wood flour as a filler within a Polylactic Acid (PLA) matrix, are among the most established and commercially available NFC filaments for FFF. Their popularity stems largely from their unique aesthetic qualities, which impart a wood-like appearance and texture to printed parts, and their ability to lower the overall cost of the filament material. However, while WPCs were historically adopted for their aesthetic qualities and cost-reduction potential, contemporary research is increasingly focused on engineering their unique property profiles, positioning them as functional materials rather than just low-cost substitutes [11,183,184]. Figure 9 provides an example of this, showing a realistic wood texture printed directly onto a substrate.

However, a comprehensive review of the literature reveals that their mechanical performance is generally subordinate to that of the neat polymer matrix. Multiple studies consistently report that the addition of wood flour to PLA results in a composite with notably lower strength. For instance, one study found that printed WPC parts with 30–40% wood content exhibited significantly lower tensile strength (4.8–7.3 MPa) and Charpy impact strength (2.9–3.3 kJ/m^2^) compared to parts made of pure PLA, which achieved 26.8 MPa and 5.4 kJ/m^2^, respectively, under the same printing conditions. This performance profile renders WPC parts suitable primarily for non-structural, low-stress applications such as architectural models, decorative items, and visual prototypes where mechanical robustness is not the primary requirement. A recurring observation in the study of WPCs is a distinct mechanical trade-off. While tensile strength decreases, the stiffness (Young’s Modulus) can either increase or decrease compared to pure PLA, depending on the filler content. For example, composites with a high wood concentration (30–40 wt%) have shown a decrease in tensile modulus compared to neat PLA. Conversely, other research demonstrates that a lower filler concentration (20 wt%) can effectively increase the Young’s Modulus of printed parts to 2600–3100 MPa, outperforming pure PLA. This reinforcing effect is highly dependent on filler loading, as some studies indicate that tensile strength may slightly increase at very low wood concentrations (e.g., 10 wt%) before decreasing significantly at higher levels. This suggests the wood flour acts more as a stiffening “functional filler” rather than a true “reinforcement” for strength [11,184,189,190,191].

When a tensile load is applied, micro-cracks initiate at these weak particle-matrix interfaces, propagating through the material and causing it to fail at a lower overall stress than the more homogenous, defect-free pure polymer. The primary purpose of its inclusion is to modify aesthetics and increase stiffness, not to create a high-strength structural composite. This distinction is critical for managing application expectations and guiding research toward strategies that can achieve genuine reinforcement. The final properties of FFF-printed WPCs are highly sensitive to both material composition and processing parameters. Research has shown that using finer wood flour particles, for instance, those smaller than 125 µm, can improve both tensile and flexural strength while also reducing water absorption compared to composites made with coarser particles [183,189,193]. Performance enhancements are typically observed only at low filler concentrations, generally below 10 wt%, with higher fiber content leading to a degradation of mechanical properties. FFF process parameters also play a critical role, for example, lower printing speeds (e.g., 30 mm/s) have been shown to produce parts with higher density, better surface finish, and superior compressive properties compared to higher speeds (e.g., 70 mm/s), which can result in uneven surfaces and a greater incidence of fiber pull-outs on fracture surfaces. Furthermore, material modification strategies can yield improvements. Thermally modifying the wood particles before compounding has been shown to enhance interfacial adhesion with the PLA matrix, resulting in higher composite strength compared to that with unmodified wood. Post-processing techniques, such as heat treatment of the printed part, can also be employed to improve surface quality and hardness [190,194,195]. Like other NFCs, WPCs are susceptible to environmental degradation. Exposure to ultraviolet (UV) radiation, particularly in combination with elevated temperatures, causes significant deterioration. This degradation manifests visually as discoloration (whitening or yellowing) and structurally as severe internal damage and porosity, leading to tensile strength reductions of as much as 44%. Similarly, hydrothermal aging results in surface whitening and has a negative impact on both tensile properties and the overall thermal stability of the composite [196,197].

#### 5.2.2. Flax-Reinforced Composites

Flax fibers are recognized for their attractive specific mechanical properties, making them a promising candidate for reinforcing thermoplastic polymers like PLA in FFF applications. The performance enhancement is highly dependent on the form of the fiber reinforcement.

Studies focusing on short flax fibers (SFF) show that their addition can significantly increase the stiffness of PLA-based composites. For example, incorporating 20–40 wt% of SFF has been shown to increase the tensile modulus from approximately 3300 MPa to a range of 6500–7300 MPa, an increase of 88–121%. An optimal tensile strength is often achieved at a specific loading, with one study reporting a peak tensile strength of 66 MPa at a 20 wt% loading slightly exceeding that of neat PLA (65 MPa) before decreasing at higher fiber concentrations. The reinforcing effect is far more pronounced when using continuous flax fibers (CFF). Research on 3D printed CFF composites has demonstrated remarkable improvements, achieving tensile strengths of up to 253.7 MPa and a tensile modulus as high as 23,300 MPa. This represents a more than four-fold increase in strength and a seven-fold increase in stiffness over neat PLA. However, realizing this potential is highly contingent on achieving strong interfacial adhesion between the fiber and matrix, a goal that often necessitates the use of fiber surface treatments or coupling agents. However for mechanical enhancement, flax-PLA composites are particularly vulnerable to environmental degradation, which is a critical limiting factor for their long-term durability [198,199,200].

**Figure 10:**
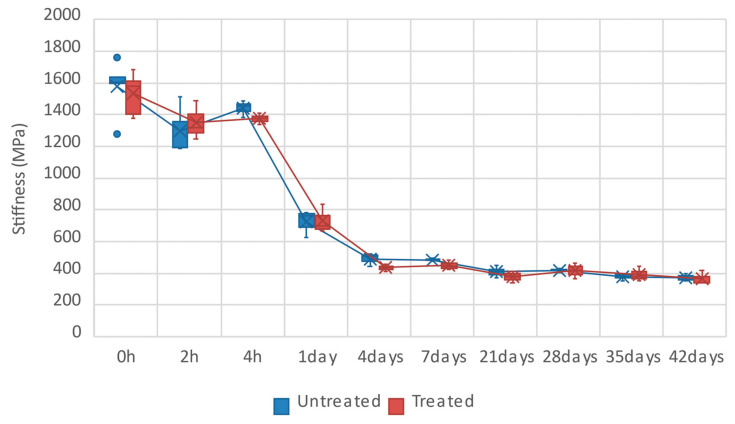
The effect of hygrothermal aging on the stiffness of flax-PLA composites. The graph highlights a critical vulnerability and technical conclusion: the material experiences a significant loss of stiffness within the first 24 h of exposure. This rapid degradation is driven by water absorption and damage to the fiber–matrix interface, demonstrating the poor long-term durability of these composites in humid environments [201].

Long-term immersion in water or exposure to hygrothermal conditions, such as 40 °C with water vapor, leads to severe degradation of mechanical properties. Experiments involving immersion in seawater at 40 °C for a period of nine months have demonstrated a dramatic reduction in tensile strength by up to 60%. This degradation is a complex, multi-faceted process involving the hydrolytic degradation of the PLA matrix, progressive damage to the fiber–matrix interface, and plasticization effects from water absorption, all of which compromise stress transfer. The degradation often proceeds in stages, with stiffness being the first property to decline significantly as demonstrated by the sharp drop within the first 24 h of exposure shown in Figure 10 while a reduction in ultimate strength follows over a more extended period of several weeks [189,198,201,202,203].

Exposure to UV radiation presents another significant degradation pathway. Weathering tests simulating sunlight exposure have shown a consistent decline in the mechanical properties of flax composites. For example, 1500 h of weathering can result in a 17% reduction in tensile strength and a 38% reduction in tensile modulus. This photodegradation is typically concentrated near the surface of the composite and can be mitigated to some extent through the use of inorganic fillers that block UV light or by applying protective surface films. The dynamics of moisture absorption are a key driver of these failure mechanisms. The hydrophilic nature of flax fibers causes them to swell as they absorb water, which generates significant micro-stresses at the fiber–matrix interface, leading to the formation of cracks and delamination. Interestingly, this interaction produces a nuanced effect on mechanical behavior: while moisture absorption generally leads to a decrease in stiffness and yield stress, some studies have reported that it can simultaneously increase the impact energy absorption capability of the composite. This makes the material tougher and less prone to brittle fracture due to the plasticizing effect of water, which allows for greater energy dissipation prior to failure, albeit at the cost of rigidity. The pronounced degradation of flax-PLA composites in marine environments raises important questions about their “green” credentials. While these materials are often promoted as sustainable “bio-composites” and have been considered for marine applications, their rapid degradation in seawater presents a significant environmental concern. Studies on the leachates from these degrading composites may show no immediate chemical toxicity to marine organisms like copepods, this analysis overlooks the significant physical hazard posed by the generated microplastic pollution [17,201,203,204,205,206].

#### 5.2.3. Hemp-Reinforced Composites

**Figure 11:**
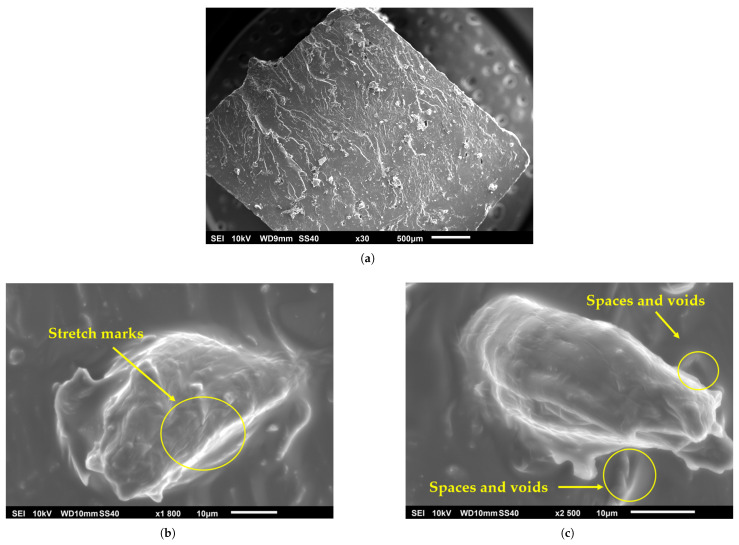
SEM micrographs illustrating the poor interfacial adhesion between a hemp fiber and the polymer matrix in a composite. (**a**) Low-magnification (×30) overview of the fracture surface. (**b**) High-magnification (×1800) view showing stretch marks on the fiber surface. (**c**) Higher-magnification (×2500) view clearly indicating a weak bond, evidenced by the presence of spaces and voids at the fiber–matrix interface [207].

The effect of untreated hemp fibers on the mechanical performance of PLA composites is complex, with conflicting results reported in the literature. Some studies show a characteristic trade-off, with tensile strength decreasing from approximately 60–70 MPa for neat PLA to a range of 35–45 MPa for composites, while the tensile modulus increases from 3400 MPa to over 4100 MPa. representing a stiffness increase of approximately 23%. However, other research reports either a much more severe drop in strength or even an increase in tensile strength at low fiber loadings. This variability highlights the sensitivity of the composite’s performance to factors such as fiber quality, processing conditions, and interfacial adhesion [189,207,208]. This reduction in strength is primarily caused by poor interfacial adhesion between the untreated hydrophilic fibers and the hydrophobic polymer matrix, a phenomenon clearly visible in the SEM micrographs in Figure 11.

True potential of hemp as a reinforcing agent can be unlocked through assumed chemical modification of the fiber surface, a widely employed strategy to improve fiber–matrix bonding. Research has demonstrated that such treatments can dramatically improve the mechanical properties of hemp–PLA composites. For example, one study showed that applying a cationization treatment to hemp fibers significantly improved interfacial adhesion, leading to an increase in the composite’s tensile strength by as much as 615% relative to the untreated composite. While this enhancement transformed the hemp from a strength-degrading defect into a functional reinforcement, the resulting tensile strength was still moderately lower than that of pure PLA. However, the study further demonstrated that combining the treated fibers with a compatibilizer (GMA) recovered the tensile performance to a level approximately 7% below that of the neat polymer matrix [207,208,209]. This finding underscores a critical principle for NFCs: their performance is not limited by the intrinsic properties of the fibers alone but is heavily dependent on the engineering of the interfacial region, which governs stress transfer from the matrix to the reinforcement. Beyond material composition, the final properties of FFF-printed hemp–PLA parts are heavily influenced by the printing process itself. The choice of infill pattern has been identified as a particularly critical parameter. While unidirectional patterns (0° raster angle) are theoretically strongest along the testing axis, one study found that specimens printed with a cross-hatch infill pattern (±45° raster angles) exhibited superior tensile performance. This counter-intuitive result suggests that process-induced defects, such as poor fusion between adjacent parallel print lines, can negate the theoretical advantages of unidirectional reinforcement, and that cross-ply patterns may offer more robust performance under non-optimal printing conditions. This is because the alternating layers of the cross-hatch pattern provide more effective load distribution and create a structure that is more resistant to delamination under tensile stress. As with other NFCs, FFF-printed hemp–PLA composites are susceptible to accelerated aging, with measurable degradation and mass loss observed within the first week of exposure to aging conditions. The highly hygroscopic nature of hemp fibers is the primary driver of this degradation, reinforcing the necessity of chemical treatments not only for initial mechanical performance but also for ensuring long-term environmental stability. The highly hygroscopic nature of hemp fibers is the primary driver of this degradation, reinforcing the necessity of chemical treatments not only for initial mechanical performance but also for ensuring long-term environmental stability [19,189,209].

#### 5.2.4. Jute-Reinforced Composites

**Figure 12:**
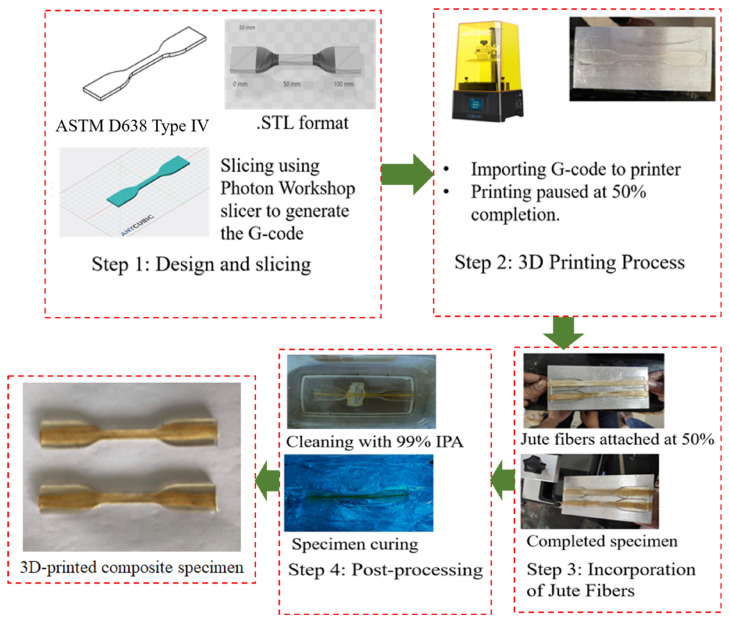
A schematic of an alternative additive manufacturing (AM) process for natural fiber composites: stereolithography (SLA) with mid-print jute fiber incorporation. The key technical conclusion is that the manufacturing method is a critical variable for composite performance. This SLA-based approach (Steps 2–3) demonstrates a promising alternative to traditional fabrication, which can suffer from high void content, and to FFF, which faces rheological challenges. The process shown here, by embedding fibers directly into the liquid resin, aims to achieve superior impregnation and fiber–matrix adhesion, which has been reported to significantly enhance mechanical properties compared to the pure resin matrix [212].

Among the various natural fibers investigated for FFF, jute is recognized as a particularly promising reinforcement due to its high cellulose content (45–71.5%), favorable specific strength, biodegradability, and low cost. However, the performance enhancement is highly dependent on the form of the reinforcement. For instance, a seminal study on FFF-printed composites utilizing continuous jute fibers reported remarkable improvements over pure PLA, with tensile strength increasing by 134% increase in tensile strength to 57.1 MPa, and a 157% increase in Young’s modulus to 5110 MPa. Conversely, when short, pulverized jute fibers are used, a different mechanical trade-off is often observed where the fibers act as stress-concentrating defects rather than reinforcement. For example, one study found that adding 5 wt% of pulverized waste jute to a PLA matrix decreased the tensile strength of FFF-printed specimens from 35.2 MPa for neat PLA to approximately 32 MPa for the composite In other additive manufacturing contexts, such as stereolithography (SLA), the use of discontinuous jute fibers has shown significant promise. One study demonstrated an approximately 84% enhancement in tensile strength, increasing from 20.4 MPa for the pure resin matrix to 37.6 MPa for the jute-reinforced composite These results position jute as a leading candidate for creating high-performance, bio-based components, as additive manufacturing can offer distinct advantages over traditional fabrication methods by enabling greater process control. Conventional techniques such as hand lay-up are often plagued by process-induced defects, particularly high void content, which can be as high as 30%. These voids act as stress concentrators and weak points, promoting premature crack propagation and significantly weakening the final part. While the precise material deposition of additive manufacturing offers a pathway to mitigate these large-scale voids, Fused Filament Fabrication (FFF) of natural fiber composites still faces significant challenges, including process-induced porosity and inconsistent material flow [210,211,212,213,214]. In contrast, alternative AM technologies such as stereolithography (SLA) have been shown to effectively reduce void content and improve fiber–matrix bonding for jute-reinforced resins, as demonstrated in the schematic shown in Figure 12.

Despite its high potential, jute-PLA composites remain highly susceptible to hygrothermal aging, a critical consideration for their long-term durability. The degradation process is highly sensitive to temperature, accelerating significantly at elevated temperatures, particularly those approaching or exceeding the glass transition temperature ($T_{g}$) of the PLA matrix (around 60 °C). The degradation mechanism is driven by moisture absorption, which initiates a cascade of detrimental effects. For example, studies on injection-molded composites have shown that while the initial strength of the composite may be comparable to the neat polymer, it degrades much more rapidly under hygrothermal conditions. These include the severe hydrolytic degradation of the PLA matrix, which breaks down the polymer chains and reduces molecular weight, and the formation of microcracks and delamination at the fiber–matrix interface due to fiber swelling. Consequently, the tensile and flexural properties of the composite decline rapidly under these conditions, although impact properties have been observed to be less severely affected in some cases [186,215].

#### 5.2.5. Overarching Challenges and Mitigation Strategies in FFF of NFCs

The fundamental challenge of weak interfacial adhesion is overcome by chemically engineering the bond between the hydrophilic fibers and the hydrophobic matrix. This is achieved through various techniques, including the application of coupling agents like Maleic Anhydride-grafted Polypropylene (MAPP) or silanes, the direct surface modification of fibers via treatments such as cationization, and the addition of compatibilizers to the polymer blend to ensure effective stress transfer [184,189,209].

To enhance long-term durability against environmental factors, protective strategies are twofold. Moisture resistance is improved by applying hydrophobic coatings or using fiber treatments that limit water uptake, while photodegradation from UV exposure is mitigated by incorporating stabilizing additives into the matrix or applying protective surface films [183,186,197,206,215,216,217,218,219].

Finally, successful fabrication requires navigating the narrow processing window defined by the composite’s high melt viscosity and the low thermal stability of the natural fibers. This is managed through a dual approach: modifying the material itself to reduce melt viscosity and meticulously optimizing FFF parameters, such as lowering print speeds and fine-tuning temperatures, to ensure complete layer fusion without causing thermal degradation [10,21,101,102,220,221,222].

Analogously, Table 3 provides a collective comparison of the mechanical properties of PLA-based composites reinforced with selected natural fibers, allowing for a quick assessment of their effectiveness as reinforcement.

## 6. Conclusions and Future Perspectives

This review generally concerns the development of 3D printing. The brief history of the creation of 3D printers is discussed at the beginning of the review. Next, the materials used in fused filament fabrication 3D printing technology are presented, namely polylactic acid (PLA), acrylonitrile butadiene styrene (ABS) and polyethylene terephthalate glycol (PETG). In the case of each material their mechanical properties, processing behavior during the printing process, thermal aging, degradation pathways, and finally influence of the UV radiation on the parts created with the use of FFF technology is discussed in detail. As shown in Table 1, the highest tensile strength, stiffness and elongation values are observed for PLA. On the other hand, the other parameters (extrusion temperature, printing speed and layer height) are comparable for all the materials studied. All of the presented FFF materials reveal significant sensitivity to the ageing phenomenon, particularly to thermal ageing. This process leads to a significant reduction in mechanical properties. Furthermore, the negative impact of UV radiation on structures created using FFF technology should be emphasised.

The second part of the review focuses on recent advances in FFF technology, specifically the addition of reinforcement to FFF materials. Typically, reinforcement is achieved using synthetic or natural short fibres. According to Table 2, the addition of carbon, glass or aramid fibres generally improves mechanical performance. For example, carbon fiber can increase stiffness by up to 700%, while continuous aramid fibers can enhance tensile strength by over 10-fold. This quantitative leap transforms these materials from prototyping tools to viable options for industrial applications requiring high strength-to-weight ratios, such as in functional automotive or aerospace components and custom manufacturing jigs. However, the ultimate effect depends heavily on the combination of different parameters. In contrast to synthetic fibers, the addition of natural fibers such as wood–plastic, flax, hemp, or jute presents a more complex trade-off. While continuous natural fibers show high potential (e.g., continuous jute increasing tensile strength by 134%), composites with short natural fibers (like wood) often act as fillers, decreasing strength (e.g., by 70–80% for WPCs) but sometimes increasing stiffness. Their primary impact lies in enabling sustainable, low-cost, or aesthetically driven applications where high mechanical load is not a prerequisite, such as in architectural models or consumer products, as shown in Table 3, and can sometimes even cause it to decrease.

However, it should be noted that the problem of the improvement in the mechanical properties, resistant to the impact of aging, environmental conditions and UV radiation by supplementing FFF materials of other ingredients, is still open.

Building on this, it is worth noting that FFF technology, and 3D printing in general, is still being dynamically developed. Three main directions for further advances can be highlighted, namely the following:

1. Hybrid manufacturing combines traditional 3d printing technology with subtractive manufacturing methods such as machining (CNC) to create parts with high precision, complex shapes, and improved properties. These technologies can be combined in a single device, where 3D printing creates the part and a CNC machine precisely finishes it or adds intricate details. The potential advantage of such an approach is high precision, impossible to achieve with 3D printing alone. Using a hybrid method can reduce costs because the main part can be printed and then machine only key surfaces or produce only the required sections from more expensive materials. Finally, a significant cost reduction can be achieved because, for short runs and on-demand production, hybrid methods avoid the need for expensive molds, such as those used for plastic injection molding.
2. In situ monitoring in 3D printing involves monitoring the 3D printing process in real time using various sensors and cameras to ensure quality and identify problems such as layering errors, deformations, or material irregularities. This provides continuous monitoring of the printing process, allowing for immediate intervention if any irregularities are detected, which is particularly important in industrial and critical applications where precision and repeatability are crucial. Monitoring can be realized with the use of different sensors, like cameras, thermovision, temperature, pressure, or ultrasonic sensors. The latter ones allow the real-time detection of different cracks and flaws, which can appear during the printing process.
3. It should be noted that the 3D printing process is not stable. Therefore, it seems that involving artificial intelligence or machine learning, which would automatically supervise in real time the process of 3D printing, seems to be very promising. For example, if the material is not being applied evenly, it can immediately adjust the speed or temperature, significantly impacting the quality of the final product. Moreover, the use of such a tool allows us to see the potential problems before they occur. AI can also be utilized in the other way, namely for analyzing the trends in designing 3D printers, allowing the creation of models that are more optimized. Such an approach not only increases the precision of the new devices but also accelerates the entire innovation process.

## Figures and Tables

**Table 2:** Comparison of mechanical properties for FFF composites reinforced with synthetic fibers.

Property	Carbon Fiber (CF) [60,139,140,142,143,149,150,151]	Glass Fiber (GF) [139,157,158,159,160,161]	Aramid Fiber [164,165,166,167]
**Young’s** **Modulus** **Improvement**	Increases from +160% (PLA-CF) up to +700% (ABS-CF) compared to neat polymers have been reported.	Increases up to +68% increase in tensile modulus reported for SGF-reinforced ABS.	Over 15-fold (∼+1400%) increase for PETG composites with continuous fibers (45 vol%).
**Tensile** **Strength** **Improvement**	Increases range from +14–47% (PLA-CF) to +22.5–33% (ABS-CF).	Increases of +31% to +57% reported for SGF-reinforced ABS composites.	Increases over 10-fold (∼+900%) increase for PETG composites with continuous fibers.
**Impact** **Resistance**	Generally exhibits brittle fracture.	Increases up to +54% increase in Izod impact strength (SGF). Up to +460% increase in Charpy impact energy for woven fabric in PLA+.	Characterized by high energy absorption and a ductile, non-catastrophic failure mode. Hybrids with aramid can increase energy absorption by 5.5–11.6%.
**Primary** **Challenge**	High cost, extreme nozzle abrasion, and brittle failure mode.	Lower absolute stiffness and higher density compared to carbon fiber.	Pronounced hygroscopicity. Challenges in achieving strong fiber–matrix adhesion.

**Table 3:** Comparison of mechanical properties for FFF composites reinforced with selected natural fibers (PLA matrix).

Property	Wood–Plastic (WPC) [11,184,189,190,191]	Flax Fiber [198,199,200]	Hemp Fiber [189,207,208]	Jute Fiber [210,211,212,213,214]
**Tensile** **Strength**	**Decrease:** 4.8–7.3 MPa (vs. 26.8 MPa for neat PLA with 30–40% wood content).	**Increase (Continuous):** Up to 253.7 MPa (∼4× increase). **No change (Short):** Peak at 66 MPa (vs. 65 MPa for PLA).	**Decrease (Untreated):** 35–45 MPa (vs. 60–70 MPa for PLA). Performance can be improved with chemical treatment.	**Increase (Continuous):** To 57.1 MPa (+134%).
**Young’s** **Modulus**	**Load Dependent:** Can increase to 2600–3100 MPa at 20 wt% content.	**Increase:** 6500–7300 MPa (+88–121%) for short fibers; up to 23,300 MPa (∼7× increase) for continuous fibers.	**Increase:** To over 4100 MPa (+120% vs. ∼3400 MPa for PLA).	**Increase (Continuous):** To 5110 MPa (+157%).
**Impact** **Resistance**	**Decrease:** 2.9–3.3 kJ/m^2^ (vs. 5.4 kJ/m^2^ for neat PLA).	-	-	-
**Primary** **Challenge**	Acts more as a filler than a reinforcement, significantly decreasing strength.	High susceptibility to degradation from moisture and UV radiation (e.g., up to 60% strength loss in seawater).	Very poor interfacial adhesion without chemical treatment, leading to high variability in results.	Large performance gap between short fibers (property decrease) and continuous fibers (property increase). Susceptible to hygrothermal aging.

## Data Availability

No new data were created or analyzed in this study. Data sharing is not applicable to this article.
